# Corrosion and Biocompatibility Studies of Bioceramic Alumina Coatings on Aluminum Alloy 6082

**DOI:** 10.1021/acsami.5c00532

**Published:** 2025-04-18

**Authors:** Tadas Matijosius, Neringa Bakute, Juozas Padgurskas, Ausra Selskiene, Aleksej Zarkov, Asta Griguceviciene, Justina Kavaliauskaite, Arunas Stirke, Svajus Joseph Asadauskas

**Affiliations:** † Faculty of Engineering, Vytautas Magnus University (VMU), Studentu 15, Akademija, Kaunas LT 53362, Lithuania; ‡ Department of Chemical Engineering and Technology, State Research Institute Center for Physical Sciences and Technology, Sauletekio 3, Vilnius LT 10257, Lithuania; § Department of Functional Materials and Electronics, State Research Institute Center for Physical Sciences and Technology, Sauletekio 3, Vilnius LT 10257, Lithuania; ∥ Department of Characterisation of Materials Structure, State Research Institute Center for Physical Sciences and Technology, Sauletekio 3, Vilnius LT 10257, Lithuania; ⊥ Department of Electrochemical Material Science, State Research Institute Center for Physical Sciences and Technology, Sauletekio 3, Vilnius LT 10257, Lithuania; # Institute of Chemistry, Vilnius University, Naugarduko 24, Vilnius LT-03225, Lithuania

**Keywords:** porous Al_2_O_3_, simulated body fluid, cell adhesion, viability, osteosynthesis plates

## Abstract

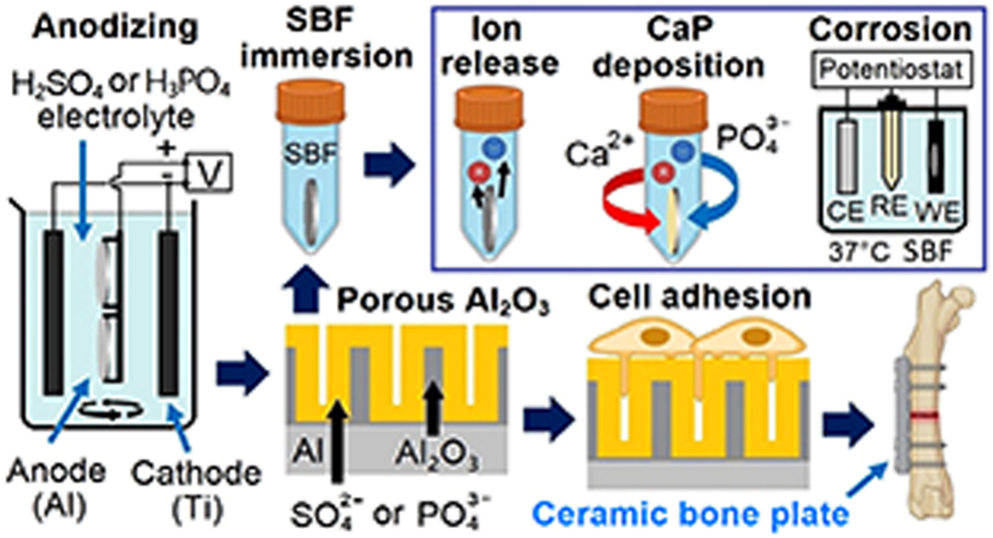

Recent advances in ceramic materials, particularly porous alumina (Al_2_O_3_), have significantly enhanced the safety and efficacy of medical implants by improving biocompatibility and modulating cellular behavior for biomedical applications. Variations in the surface structure and chemical composition of porous Al_2_O_3_ promote different biological responses and coating stability, underscoring the need for further biological and corrosion research. Traditional methods for producing alumina ceramics from powder are expensive, time-consuming, and limited in their ability to create complex shapes and large structures due to the brittleness of alumina. This study evaluates the biocompatibility of bioceramic-coated aluminum (Al) alloy 6082 as a lightweight and cost-effective alternative for bone osteosynthesis plates. Al_2_O_3_ coatings were achieved through anodization using phosphoric and sulfuric acids. The untreated and anodized alloys were analyzed for chemical stability and biocompatibility and compared with medical-grade titanium alloy. All specimens exhibited excellent biocompatibility, demonstrating high adhesion and viability of the fibroblast cell line. Corrosion resistance and metal ion release were assessed in simulated body fluid, with all specimens effectively suppressing the release of Fe and toxic Al ions. The untreated Al alloy exhibited a higher release of Mn ions than the coated specimens. Notably, the bioceramic coating obtained in sulfuric acid demonstrated 3 orders of magnitude higher corrosion resistance, indicating its potential suitability for biomedical applications. By addressing the limitations of traditional alumina ceramics, our approach enables the fabrication of products in diverse sizes and shapes, offering a practical solution for creating customized biomedical implants.

## 1 Introduction

A significant aspect of regenerative medicine is the utilization of bioceramics as biomaterials across various fields, including dental implantology, orthopedic implantology, bone grafts, and scaffolds.^1,2^ Bioceramics are defined as ceramic materials or metal oxides that demonstrate compatibility with biological systems. To effectively replace the musculoskeletal component of the human body, biomaterials must exhibit distinct and specific properties. These properties include outstanding fatigue and tensile strength, high resistance to corrosion and wear, a low modulus of elasticity, and good hardness. Additionally, they should possess a low density and demonstrate high biocompatibility. These characteristics are essential for ensuring the effectiveness and safety of biomaterials in biological systems.^3^ Bioceramics are characterized by their combination of excellent strength, high hardness, toughness, and outstanding corrosion and mechanical resistance.^4−6^ Based on their interaction with adjacent tissues, bioceramics can be classified into three categories: bioinert (alumina Al_2_O_3_ and zirconia ZrO_2_), bioactive (hydroxyapatite, and biodegradable (tricalcium phosphate) materials).^7^

Bioinert alumina Al_2_O_3_ has been utilized since 1960 as a favorable candidate in orthopedic joint prostheses due to its great compressive resistance and chemically bioinert properties.^8−11^ Al_2_O_3_ does not facilitate bonding with living tissues or body fluids, exhibits resistance to low pH environments for thousands of hours, and demonstrates high chemical inertness, indicating that a significant amount of time is required to establish stable connections between implants and tissues.^12^

While discussing the features of Al_2_O_3_, it is important to highlight that alumina Al_2_O_3_ is not the same as aluminum (Al) or Al alloy. Al is a highly reactive metal that is considered incompatible with biological systems. It can induce DNA damage, oxidative stress, disrupt cellular energy metabolism, and influence neurodegenerative diseases, neurotoxicity, cytotoxicity, and carcinogenesis.^13,14^ The surfaces of both Al and its alloys are naturally covered with a thin 2–3 nm layer of Al_2_O_3_ due to its reactivity with oxygen.^15^ However, these Al_2_O_3_ layers are too thin, resulting in inadequate mechanical properties and corrosion resistance, as well as the release of toxic Al^3+^ ions.

Porous anodic coatings with a thickness of 100 μm or more, primarily composed of bioceramic Al_2_O_3_, are obtained by anodizing Al alloy in acidic electrolytes.^16^ Al_2_O_3_ coatings consist of long vertical nanostructured arrays featuring a hexagonal pore structure, with open nanopores on the top side, that terminate in a thin nonporous barrier layer next to Al. The thick Al_2_O_3_ layer enhances surface hardness, mechanical resistance, corrosion resistance, and other technical properties. Nanoporous Al_2_O_3_ exhibits good biocompatibility with neuronal, epithelial, muscle, connective tissue, and blood cells.^17^ Surface pore parameters can influence cell adhesion and proliferation. For instance, pores with a size of 75 nm have been shown to promote higher levels of cell adhesion and proliferation compared to larger pores, owing to the increased focal adhesion densities.^18^ Higher surface nanoporosity also enhances fibroblast migration, attributed to larger focal adhesions, aligned actin fibers, and polarized cell morphology.^19^ Surface texturing of bioceramic Al_2_O_3_ indicates improved biocompatibility and greater control over cellular behavior for biomedical implants.

Cell adhesion and biocompatibility can be enhanced by using nanoporous Al_2_O_3_ structures.^20,21^ However, it is important to note that the biological response may vary depending on the composition of the Al alloy and the anodizing electrolyte. This variation arises not only from the release of metal ions but also from changes in surface chemistry. Depending on electrolyte composition, porous Al_2_O_3_ can incorporate small amounts of anions, such as up to 14 wt % sulfates, up to 8 wt % phosphates, and up to 3 wt % oxalates.^22^ Phosphates are indispensable biomolecules that play a crucial role in numerous biological processes, including energy metabolism, cell signaling, regulation of protein synthesis, and skeletal mineralization.^23^ In contrast, the presence of sulfates, in combination with Al, has been linked to a range of adverse effects, including increased oxidative stress, inflammation, cytotoxicity, neurotoxicity, genotoxicity, and other negative reactions.^24,25^ It is noteworthy that sulfate-based electrolytes are commonly utilized in the production of thick nanoporous Al_2_O_3_ coatings, which enhance surface hardness and mechanical resistance. Phosphate electrolytes, on the other hand, produce surfaces with pores exceeding 100 nm in diameter. Therefore, when assessing the biocompatibility of bioceramics, it is imperative to consider not only the material composition but also the surface chemistry and topography.

Alumina ceramics for implants are typically made from powder. The production process usually involves several demanding steps, including mechanical grinding, dry pressing, sintering, and extrusion at high temperatures and pressure.^26^ This manufacturing process is expensive and time-consuming, and it is limited in its ability to produce complex shapes and large structures due to the brittleness of alumina, as well as impurities and variations in composition. In contrast, our paper describes the formation of an Al_2_O_3_ coating on the lightweight Al alloy itself, enabling the fabrication of customizable products in diverse sizes and shapes. The samples obtained could serve as an alternative to the heavy Ti alloys currently used in regenerative medicine.

The quest to enhance the success rate of bone implantation has led to significant advancements in bioceramic coatings, which play a crucial role in ensuring chemical stability and promoting favorable biological responses. In this study, we demonstrate that the bioceramic coating of lightweight aluminum alloy 6082 may provide a cost-effective alternative to the expensive bone osteosynthesis plates currently in use. By analyzing the untreated Al alloy 6082 and its two anodized forms—treated with sulfuric or phosphoric acid electrolytes—we aim to uncover their properties and compare them with a medical-grade titanium alloy. Our comprehensive evaluation includes assessing metal ion release and corrosion resistance in simulated body fluid (SBF), and examining biocompatibility through the analysis of fibroblast cell adhesion and viability. Through this research, we aim to gain a deeper understanding of the properties of bioceramic coatings and inspire future innovations in the field of biomedical implants.

## 2 Experimental

### 2.1 Materials

Al alloy 6082, with a purity of 96.52 wt % (2.28 wt % Mg; 0.53 wt % Si; 0.36 wt % Fe; 0.31 wt % Mn) and Ti alloy BT1, with a purity of 96.32 wt % purity (1.76 wt % Mn; 1.75 wt % Al; 0.11 wt % Fe; 0.06 wt % Si), both with a diameter of 16 mm and a sheet thickness of 2 mm were purchased from FXB-Niemet UAB (Lithuania) and UAB SP MET (Lithuania), respectively. All electrolytes used in the experiments were prepared from analytical grade reagents and deionized water. Laboratory-grade solvents were utilized for cleaning and degreasing.

### 2.2 Specimen Preparation and Characterization

Al alloy 6082 underwent electrochemical oxidation (anodizing) in H_2_SO_4_ and H_3_PO_4_ electrolytes. First, the alloy was degreased with acetone and then cleaned by immersing it in an alkaline solution (30 g/L sodium hydroxide (NaOH) + 25 g/L trisodium phosphate (Na_3_PO_4_) + 75 g/L sodium carbonate (Na_2_CO_3_)) for 45 s at 60 °C. This was followed by immersion in 10% HNO_3_ for 60 s at 21 °C, with rinsing in deionized water after each step. The prepared specimens were then placed into a titanium holder and submerged in a continuously mixed aqueous 3 L electrolyte bath with a titanium cathode. Type III hard anodizing was carried out in 18 wt % H_2_SO_4_ with 2 wt % oxalic acid and approximately 0.4 wt % Al^3+^ concentration at 15 °C and a 200 A/m^2^ anodic current density for 70 min, producing thick bioceramic Al_2_O_3_ coatings with small ∼15 nm nanopores.^27^ The oxalic acid additive was used to reduce the dissolution of the coating and to form denser and more stable Al_2_O_3_ during anodizing. The addition of oxalic acid to the sulfuric acid electrolyte can increase the thickness and hardness of the alumina coating, leading to improved corrosion resistance.^28−30^ Subsequently, phospho-anodizing was carried out in a 4 wt % H_3_PO_4_ electrolyte at 15 °C and 150 V direct current for 150 min to obtain thin coatings with wide ∼200 nm nanopores.^31^ The bath composition and operating conditions for anodizing are summarized in Table 1. After anodization, the specimens were immersed in a 170 W ultrasonic bath VTUSC3 (Velleman, Belgium) and sonicated at full power for 10 to 20 min in deionized water without heat for rinsing. The coating thickness was measured with a Dualscope MP0R-FP device (Helmut Fischer GmbH) using an amplitude-sensitive eddy current method on electrically conductive Al. Afterward, the specimens were dried at 60 °C for 60 min and stored in a desiccator. Before experiments, the specimens were sterilized with 70% ethanol, washed with sterile deionized water, and dried under UV light for 30 min.

**Table 1:** Electrolyte Composition and Anodizing Conditions of Al Alloy 6082

specimen	electrolyte composition	time	temperature	current density/voltage
Al_2_O_3_^S^	18% H_2_SO_4_	70 min	15 °C	200 A/m^2^ (∼20 V)
	2% oxalic acid			
	0.4% Al^3+^			
Al_2_O_3_^P^	4% H_3_PO_4_	150 min	15 °C	150 V

Contact angle measurements of water drop were conducted using EasyDrop (Kruss, Germany) instrument and “Drop Shape Analysis v.1.70–02” software. Dosing was conducted using a syringe pump with drop volumes of 24.7 ± 3.6 μL. The liquid was collected into a free-hanging drop that detaches due to mechanical action. A minimum of five water drops at various locations were measured, and their average was calculated to determine the actual contact angle.

The surface roughness *R*_a_ was tested using a stylus profilometer Surftest SJ-210 (Mitutoyo, Aurora, IL), which scanned the surfaces horizontally along a path length of 1.5 mm with a diamond tip needle of 2 μm radius, at least 10 scans at random locations.

Surface hardness was tested using indentation and Vickers microhardness (HV) techniques. Indentation hardness was performed with a Hysitron Ti Premier (Bruker) using a Berkovich diamond indenter with an angle of 142.3°. Average hardness was estimated by increasing the loading force in 0.1 N increments from 0.1 to 1 N at nine different points. The maximum penetration depth did not exceed 7 μm during the scans. Average hardness values were calculated using TriboScan software version 9. For Vickers hardness measurement, a PMT-3 indenter with a square-based diamond pyramid, angled of 136°, was used. Beforehand, the cross-section of the bioceramic coatings was prepared. The side designated for HV evaluation was polished with 320–2500 grade paper to achieve a smooth surface. An indenter load of 20 gf was maintained for 10 s to calculate HV (kgf/mm^2^) using the following formula
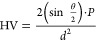
1where *P* is the load, g; *d* is the diagonal of a diamond prism, mm; θ is the apex angle of a diamond prism. The average value of HV was calculated from at least five measurements.

XRD patterns of the Al alloy and bioceramic samples were measured using a SmartLab X-ray diffractometer (Rigaku) equipped with a 9 kW rotating Cu anode X-ray tube. The grazing incidence (GIXRD) method was applied in the 2θ range of 10–80°. The angle between the parallel beam of X-rays and the specimen surface (ω angle) was set to 0.5°. Phase identification was performed using PDXL software package (Rigaku) and the ICDD powder diffraction database PDF-4+ (2023 release).

### 2.3 SBF Immersion Experiments

For *in vitro* bioactivity assessment, samples were immersed in SBF, and the formation of hydroxycarbonate apatite (HCAp) crystals on the specimens’ surface was examined. SBF was prepared with the following concentrations of cations: 142 mM Na^+^, 5 mM K^+^, 1.5 mM Mg^2+^, 2.5 mM Ca^2+^, and anions: 147.8 mM Cl^–^, 4.2 mM HCO_3_^–^, 1 mM HPO_4_^–2^, 0.5 mM SO_4_^–2^, with pH = 7.4 at 36.5 °C, following instructions provided by Kokubo and Takadama.^32^ Briefly, before use, the anhydrous materials were heat-dried and stored in a desiccator. Each component was weighed, dissolved in a small volume of water, and added to the SBF solution drop by drop in the exact order specified in the publication. Specimens with a diameter of 16 mm were immersed in 50 mL of SBF solution for 1 to 28 days at a temperature of 37 °C, maintaining the same solution for the entire duration of the experiment without renewal. Inductively coupled plasma optical emission spectroscopy (ICP-OES), pH measurements and potentiometry were conducted with the SBF, while scanning electron microscopy (SEM) – on the specimens.

### 2.4 ICP-OES

The ion release of analytes from specimens into SBF for 1 to 28 days was assessed by ICP-OES using a PerkinElmer Optima 7000 DV spectrometer. SBF samples prior to analysis were diluted 10 times with deionized water, and concentrations of Al, Fe, Mg, Mn, Si, Ti, P, and S analytes were recorded in mg/L.

### 2.5 Corrosion Tests

Electrochemical voltammetric (*j*/*E*) and impedance (*Z*) measurements were performed in SBF at 37 °C using the Solartron 1280C potentiostat/frequency analyzer system (Ametek, Inc.). The Ag/AgCl electrode in saturated KCl solution was used as a reference, and the 4 cm^2^ Pt plate as a counter electrode. The working electrode was mounted in a holder and placed into an electrochemical glass cell with ∼100 mL SBF so that the area of the electrode exposed to the electrolyte was 0.5 cm^2^. Electrochemical measurements were started 10–15 min after immersion to ensure the quasi-steady state value of open circuit potential (OCP). The electrochemical impedance spectroscopy (EIS) spectra were recorded under potentiostatic conditions in the 100 kHz to 0.1 Hz frequency range with a perturbation amplitude of ± 10 mV. It should be noted that the measurements in the low frequency range were limited to 100 mHz due to significant data scatter, which was most likely caused by significant changes in surface properties. The measured impedance data were analyzed based on the Kramers–Kronig (KK) relationship and were modeled using equivalent electric circuits (EEC) with ZSimpWin software.

### 2.6 Cell Line Handling

Cell culture studies were conducted using the mouse fibroblast L929 cell line (ATCC, CCL-107, NCTC clone 929 [L cell, L929, derivative of Strain L]). L929 cells were grown in RPMI-1640 medium (Corning, VA) supplemented with 10% fetal bovine serum (Gibco, Thermo Fisher Scientific, NY), 2 mM glutamine (Lonza, MD) and antibiotics penicillin 100 U/mL and streptomycin 100 μg/mL (Gibco) at 37 °C, 5% CO_2_ atmosphere in a humidified chamber. The culture medium was replaced or cells were passaged twice a week and always a day before an experiment. For the cell adhesion and viability experiments, the cells were detached by trypsinization (trypsin 0.025% and EDTA 0.02% solution) (Gibco) and suspended in the full medium at a concentration of 2 × 10^5^ cells/mL.

### 2.7 SEM Imaging

Cell suspensions were transferred to the surface of the specimens and cultured for 24 h in a CO_2_ atmosphere at 37 °C. For fixation, the specimens with cells were rinsed twice with phosphate-buffered saline (PBS) and subsequently fixed with 2% glutaraldehyde in PBS for 1–2 h at +4 °C for 15 min. After fixation, the specimens were dried using increasing concentrations of ethanol (50, 70, 85, 96, and 96%) with each concentration applied for 15 min. Finally, all samples were air-dried in a desiccator. Specimens without cells were washed with deionized water and air-dried for SEM imaging.

Dry cellular constructs were sputter-coated with Cr of 1–2 nm in thickness to obtain the necessary electrical conductivity and were imaged using a dual-beam Helios NanoLab 650 scanning electron microscope (FEI, Eindhoven, The Netherlands). The distribution of elements in the samples, both with and without SBF, was analyzed by energy-dispersive X-ray spectroscopy (EDS) using an INCA spectrometer with X-Max 20 mm^2^ Silicon-drift detector (Oxford Instruments, U.K.) at 20 kV.

Characteristics of surface pores were determined using the open-source image analysis program ImageJ 1.53t using SEM images at 50,000× and 150,000× magnifications. Thresholding was carried out in the black and white areas representing the pores and Al_2_O_3_ surface, respectively, which enabled the outlining of surface pores. Average nanopore density was determined by calculating the number of nanopores in 0.25 and 1 μm^2^ segment areas of the surface at three random locations. Surface porosity (%) was calculated using the ratio between the area of nanopore openings and the area of surface oxide selected for the calculation.

### 2.8 Viability Assays

Fibroblasts produce extracellular matrix components that are essential for cell adhesion, wound healing and tissue repair in both normal tissue and following biomaterial implantation. Monitoring the viability and proliferation of fibroblast cells facilitates the assessment of various substances’ effects on these functions. A standard model for evaluating the biocompatibility and toxicity of biomaterials is the L929 mouse fibroblast cell line. This cell line is widely used in research, ensuring the reproducibility and comparability of results across different studies.

The viability of L929 cells was assessed using an elution test method. This procedure involves incubating the cells with an extract of the test material. The extracts were obtained by placing the specimens in a cell culture medium and incubating them for 4 days at 37 °C. As a control, the cell culture medium was incubated without the immersion of specimens. The resulting fluid extracts were then collected and subsequently incubated with the cells.

To evaluate overall cell growth, including adhesion and proliferation, L929 cells were detached using trypsin and resuspended in extracts of the specimen. The cells were then seeded at a density of 10^5^ cells/well in a 6-well plate and incubated for 48 h. To assess metabolic activity, cells were washed with PBS and incubated with resazurin (Applichem, Darmstadt, Germany) at a concentration of 20 μg/mL in a buffer containing 7 mM NaCl, 1 mM KCl, 10 mM HEPES, 3mM NaOH, 250 mM saccharose, pH 7.4, for 30–60 min. Fluorescence measurements were obtained using a fluorescence spectrophotometer (LS50B, PerkinElmer) with excitation and emission wavelengths set at 571 and 584 nm, respectively. Each sample was prepared in duplicate, and the experiments were repeated three times. Relative cell viability was calculated as the ratio of the test specimen to the control specimen, expressed as a percentage.

To evaluate the impact on cell proliferation, extracts from the specimens were applied to a precultured cell monolayer (seeding density of 5 × 10^3^ cells/well in a 96-well plate, precultured for 24 h) and incubated for an additional 24 h. Following this incubation, measurements were obtained using the XTT kit (Roche, Mannheim, Germany) for 15–45 min, with absorbance recorded at 490 nm and a reference wavelength of 620 nm. Each sample was prepared in quadruplicate, and the experiments were conducted three times. Relative cell viability was calculated as the ratio of the test specimen to the control specimen, expressed as a percentage.

### 2.9 Statistical Analysis

All data are presented as the mean ± standard deviation (SD). A one-way analysis of variance (ANOVA) was employed for multifactorial comparisons in this study. A *P* value < 0.05 was considered statistically significant.

## 3 Results and Discussion

### 3.1 Structural Characterization of Bioceramic Coatings

This research was primary focused on the biocompatibility and corrosion studies of Al alloy and bioceramic Al_2_O_3_ coatings. Al alloy 6082, known for its relatively high strength and corrosion resistance, was selected for this study. The Al alloy was anodized using two different types of electrolytes to produce porous Al_2_O_3_ coatings: sulfuric acid and phosphoric acid. The selection of electrolytes is important for achieving nontoxic coatings with the appropriate thickness and surface porosity. Strong sulfuric acid and phosphoric acid electrolytes offer several advantages over weaker acids, such as oxalic acid. These advantages include a faster anodizing process, lower energy consumption, and reduced costs. Chromic acid, another common electrolyte, is known for its excellent corrosion resistance and minimal impact on the material’s mechanical properties. However, it contains chromium (VI) compounds, as well as smaller quantities of chromium (III). Chromium (VI) is highly toxic and carcinogenic. Although chromium (III) compounds are less toxic, they still pose some health risks.^33,34^

Bioceramic coatings exhibit exceptional adhesive bonding to the Al metal, enhancing bonding strength by up to 3.5 times.^35,30^ Unlike traditional deposition methods, bioceramic coatings form directly on the Al metal, facilitating mechanical interlocking. Following anodization, the ratio of penetration to buildup of the Al_2_O_3_ coating can range from 1/3 penetration and 2/3 buildup to a balanced distribution of fifty-fifty, which helps to avoid any delamination processes. By manipulating the size of nanopores, their density, surface porosity, and roughness, it is possible to control biocompatibility, cell adhesion, wettability, and other properties of bioceramic coatings.

**Figure 1:**
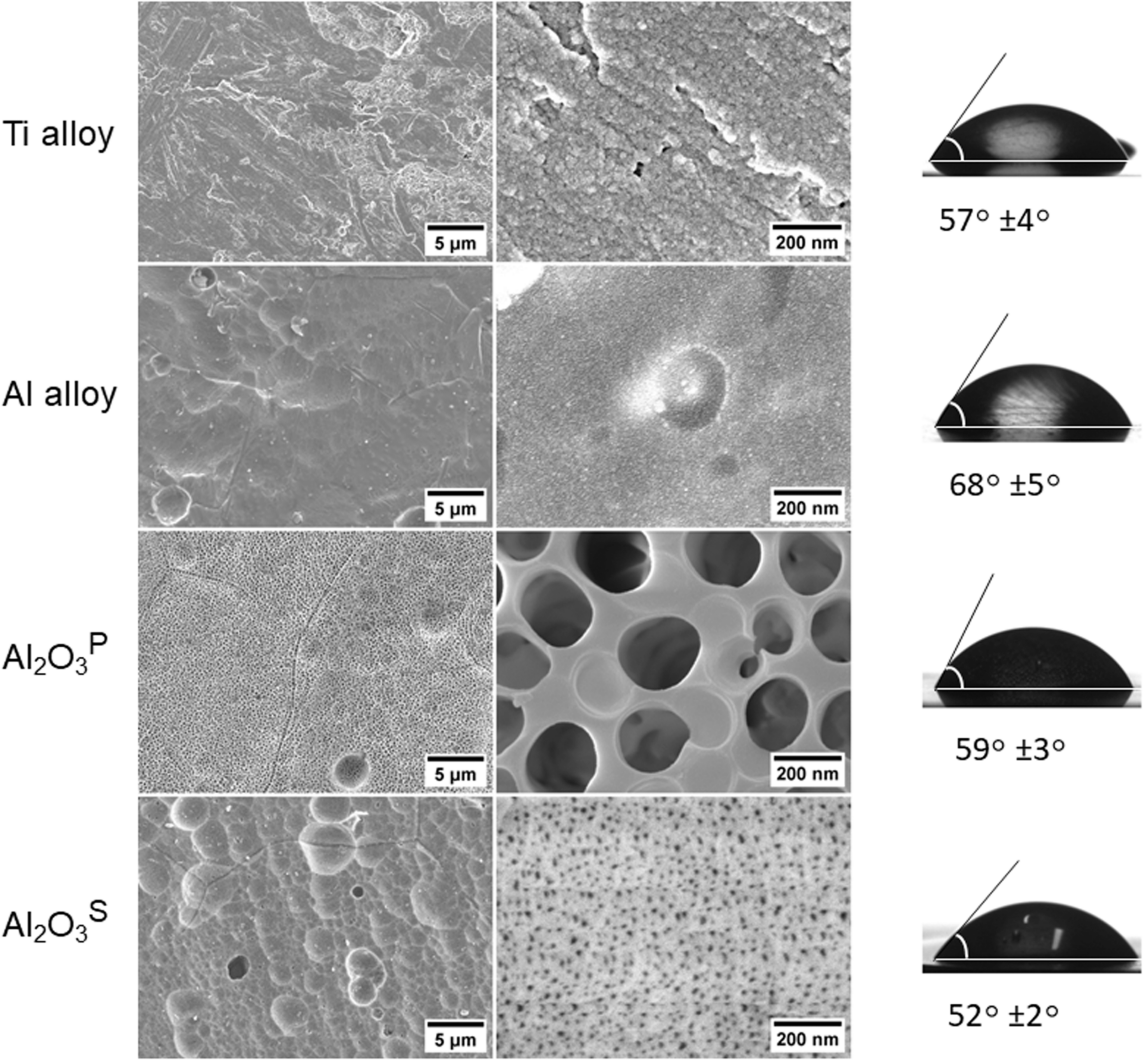
SEM topography (left) of the tested specimens under low and high magnifications and their water drop contact angles (right).

SEM nanotopography analysis revealed that anodizing in H_2_SO_4_ produced thick coatings (∼60 μm thickness) with narrow nanopores of less than 20 nm, whereas anodizing in H_3_PO_4_ resulted in thin coatings (∼10 μm thickness) with large pores of approximately 200 nm (Figure 1). For clarity, the specimens obtained using sulfuric acid are designated as “Al_2_O_3_^S^”, while those obtained with phosphoric acid are referred to as “Al_2_O_3_^P^”.

The surface porosity of Al_2_O_3_^P^ was approximately 3.5 times greater than that of Al_2_O_3_^S,^ with maximum values reaching 50 and 15%, respectively (Table 2). A detailed analysis is available in Table S1 (see Supporting Information).

**Table 2:** Characteristics of Surface Pores, Obtained after Al Anodizing in Different Electrolytes

specimen	electrolyte	coating thickness (μm)	pore diameter (nm)	pore density (pores/μm^2^)	interpore distance (nm)	porosity %
Al_2_O_3_^S^	H_2_SO_4_	58.4 ± 2.3	13.7 ± 0.1	912.0 ± 83.8	30.9 ± 4.4	13.5 ± 1.4
Al_2_O_3_^P^	H_3_PO_4_	8.7 ± 1.1	177.2 ± 15.5	19.7 ± 4.0	284.3 ± 29.2	47.6 ± 2.4

A strong 18% H_2_SO exhibiting a high dissociation constant, enhanced the dissolution of Al and accelerated the growth rate of anodic oxide, resulting in the formation of thicker and denser oxide coating (Table 3). In contrast, a weak 4% H_3_PO_4_, characterized by a low dissociation constant, produced a more hydrated and less dense oxide, leading to thinner coatings. The interpore distance was significantly greater for the Al_2_O_3_^P^ coating compared to the Al_2_O_3_^S^ coating, attributable to variation in electric field distribution, oxide formation mechanisms and electrolyte concentration. Each electrolyte is defined by a specific anodic oxidation voltage range, which correlates directly with the pore spacing of the coating.^36^ The elevated voltage and reduced ionic strength of H_3_PO_4_ resulted in considerable larger interpore distances.^37^

**Table 3:** Coating Thickness and Interpore Distance of Anodic Coatings

specimen	electrolyte	coating thickness (μm)		interpore distance (nm)	
Al_2_O_3_^S^	H_2_SO_4_	57.4	56.7	36.5	37.4
		60.2	60.3	35.6	29.4
		62.6	59.4	30.5	31.6
		56.4	55.8	29.8	27.3
		56.0	59.2	26.4	24.5
Al_2_O_3_^P^	H_3_PO_4_	9.0	9.5	276.3	295.0
		7.9	9.3	312.6	270.8
		8.5	7.7	241.3	310.2
		11.1	7.3	231.8	302.8
		9.1	8.0	288.8	313.6

The composition of the bioceramic coating is not expected to consist exclusively of Al_2_O_3_. The formation of Al_2_O_3_ involves the incorporation of anions and water molecules from the electrolyte during anodizing.^38,39^ Hydrated Al_2_O_3_ and anion complexes led to variations in mass density. EDS analysis indicated the presence of sulfur and phosphorus in the bioceramic coatings, with concentrations of 4.06 and 0.85 atom% %, respectively (Table 4). This suggests that sulfates are incorporated in much higher quantities during anodizing in the H_2_SO_4_ electrolyte. The binding of phosphates with the Al_2_O_3_ coating is lower when the H_3_PO_4_ electrolyte is used due to its lower concentration, differences in electric field, as well as faster anion diffusion and washing out of the pores. Nevertheless, the incorporation of anions plays an important role in cell adhesion and viability.

**Table 4:** EDS Analysis of Tested Specimens

specimen	Ti (atom %)	O (atom %)	Al (atom %)	Mg (atom %)	Si (atom %)	Fe (atom %)	Mn (atom %)	S (atom %)	P (atom %)
Al alloy	0	n.d.	96.60	2.57	0.51	0.17	0.15	0	0
Al_2_O_3_^S^	0	63.40	32.13	n.d.	0.41	n.d.	n.d.	4.06	0
Al_2_O_3_^P^	0	65.69	32.84	n.d.	0.62	n.d.	n.d.	0	0.85

**Figure 2:**
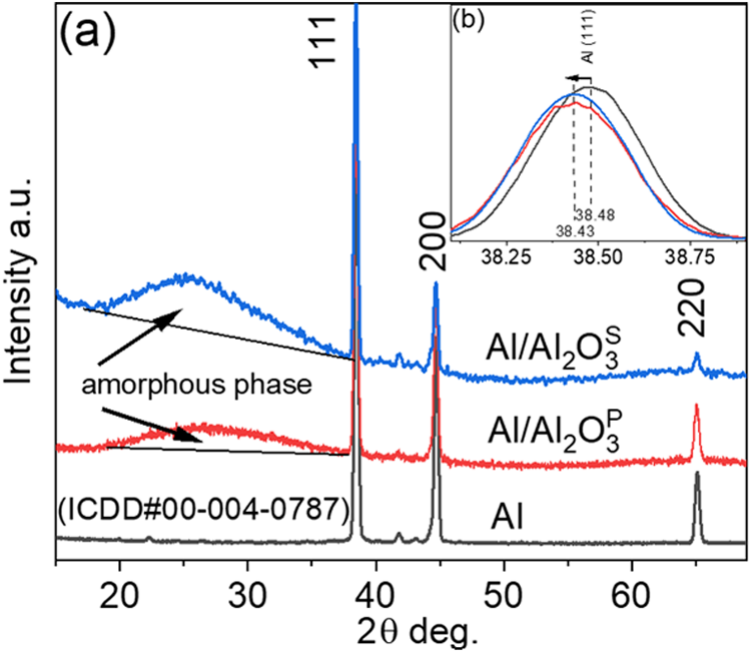
Grazing incidence X-ray diffraction (GIXRD) pattern of tested specimens (a). Pattern fragment (b) −shift of Al(111) peak after anodizing.

Surface changes of the tested specimens before and after anodizing were investigated by using the GIXRD method. The XRD profiles of Al_2_O_3_^S^ and Al_2_O_3_^P^ coatings are shown in Figure 2(a). The obtained diffraction peaks at 38.47, 44.63, and 65.13° were assigned to the 111, 200 and 220 crystallographic planes of the cubic Al phase (ICDD #00–004–0787). After anodizing, the obtained diffraction peaks (in the 2θ range of 20–30°) are very wide, indicating that the surfaces are composed of small crystallites (amorphous phase) with an average size of 1.0–3.0 nm. A more amorphous crystal surface coating is obtained on the Al_2_O_3_^S^ sample as a result of a thicker oxide layer. This can affect corrosion and surface chemical activity. Figure 2 (b) shows the shift of the Al(111) peak, after anodizing, in the direction of decreasing 2θ angles (from 38.48 to 38.43°), which indicates an increase in the parameters of the crystal lattice. The change in the parameters of the Al crystal lattice can be associated with an increase in the amount of surface defects and an increase in surface roughness.

Surface roughness is an important parameter, that can enhance the osseointegration of medical implants.^40^ Macro-roughness is directly related to implant geometry and provides initial fixation and as well as long-term mechanical stability. The combination of microscale and nanoscale roughness enhances both biological response and osseointegration.^41^ Nanoscale roughness improves cell adhesion, proliferation, spreading, and while also inducing osteogenic-related gene expression of alkaline phosphatase, osteocalcin, and osteopontin.^42^ In this study, the surface roughness *R*_a_ values of Ti and Al alloys were 1.21 ± 0.09 and 1.28 ± 0.08 μm, respectively. Anodizing might influence surface homogeneity, as the *R*_a_ values for porous Al_2_O_3_ were slightly higher, i.e., 1.45 ± 0.23 μm for Al_2_O_3_^S^ and 1.49 ± 0.14 μm for Al_2_O_3_^P^.

**Figure 3:**
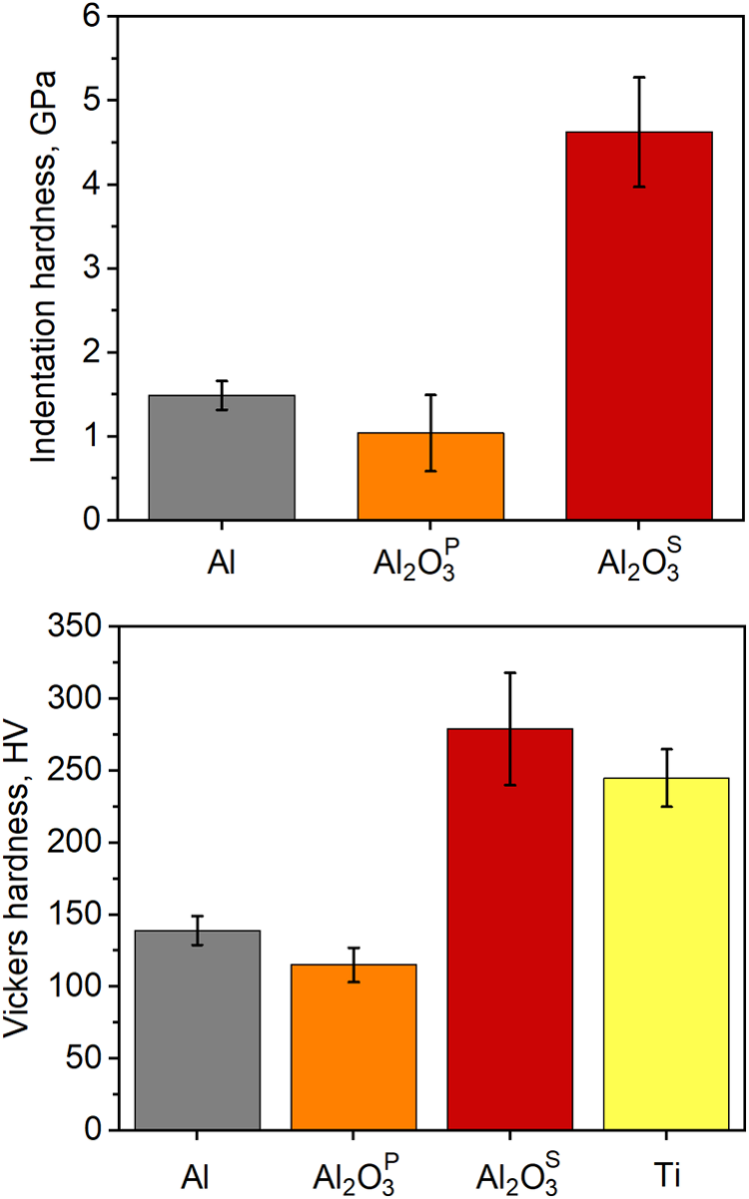
Indentation (top) and Vickers microhardness (bottom) of the tested specimens.

The mechanical studies using indentation and Vickers techniques revealed that bioceramic coatings based on Al_2_O_3_^P^ did not improve surface hardness compared to untreated Al alloy, primarily due to wide pores and high surface porosity (Figure 3). Depending on the testing method, the hardness of the Al_2_O_3_^S^ coating increased two to three times, making it comparable to that of Ti alloy. The variations in hardness may be attributed to different measurement techniques and loading conditions. The hardness of the Al_2_O_3_^S^ coating was similar to that of nanocomposite coatings based on TiO_2_/hydroxyapatite, which were developed using the sol–gel method by other researchers for medical applications.^43^

The degree of wetting, indicated by the contact angle between a liquid drop and a surface, is determined by balancing the intermolecular interactions of adhesive and cohesive forces. Surface wettability primarily depends on chemical composition, morphology, and surface roughness, and it strongly correlates with biological interactions.^44^ A water drop contact angle greater than 90° indicates a hydrophobic solid surface, which has a low surface energy and poor wetting. Conversely, a contact angle below 90° suggests better wetting and hydrophilic properties, associated with high surface energy in solids. According to Giridhar et al., surface wetting can be categorized as hydrophobic (contact angle >80°), moderately wettable (contact angle 48–62°), and hydrophilic (contact angle <35°).^45^ All tested specimens exhibited relatively hydrophilic properties, with water drop contact angles below 90° (Figure 1). The wetting properties of Al alloy were poor, displaying a water drop contact angle of 68° ± 5°. The anodized samples exhibited a range of water drop contact angles; an initial higher contact angle was recorded, which subsequently decreased and stabilized within a short duration (approximately 60 s): from 75° ± 8° to 59° ± 3° for the porous Al_2_O_3_^P^ and 60° ± 9° to 52° ± 2°, for the nanoporous Al_2_O_3_^S^. The decrease in contact angle could be related to the porous surface of the Al_2_O_3_ coating. Meanwhile, the Ti alloy exhibited a contact angle of 57° ± 4°. Biomaterials with moderate hydrophilicity (contact angle 50–80°) suggest improved cell growth and higher biocompatibility, as discussed in previous studies.^44,46^

### 3.2 Metal Ion Release

Highly porous and rough surfaces can impact the release of metal ions if the materials are not chemically pure. To evaluate the release of ions from metallic alloys and bioceramic coatings, the samples were immersed in SBF for a duration of 1 to 28 days. To ensure the experiment accurately simulates the degradation behavior of the samples in the human body, the pH of SBF should remain within a physiological range of ±7.4. Fluctuations in the pH of the SBF solution can significantly influence the degradation behavior of these specimens.^47^ For this reason, the pH of the SBF was continuously monitored throughout the immersion process.

**Figure 4:**
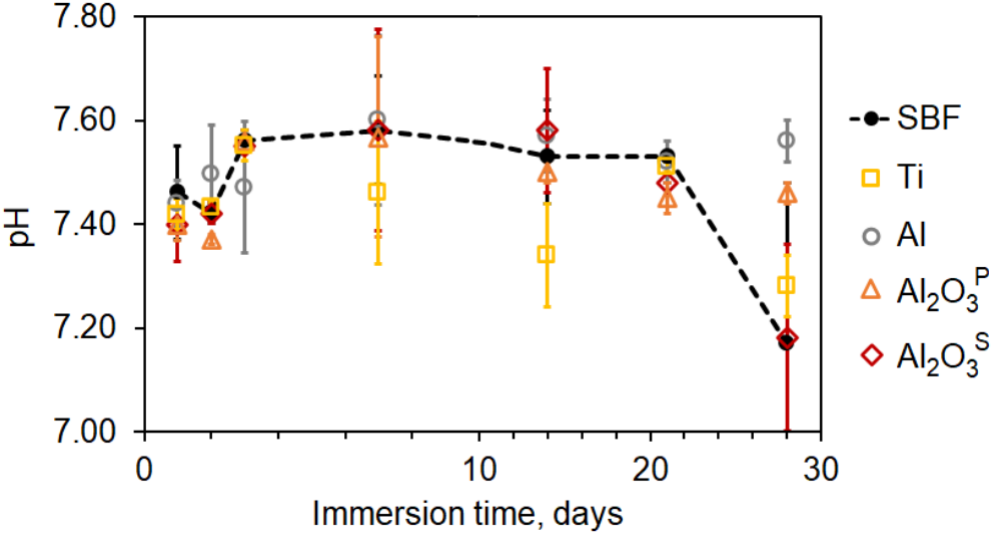
pH of SBF during the immersion experiments with the specimens.

The starting pH of SBF was 7.4 at 36.5 °C. After three days, a slight increase in pH was observed, although it did not exceed 7.50 (Figure 4), which does not interfere with physiological conditions.^48^ Significant changes were noted only by the 28th day of the experiment, when the pH of plain SBF and Al_2_O_3_^S^ decreased to 7.29. By the conclusion of the immersion experiment on the 28th day, the SBF buffer was nearing its expiration date, which may account for the observed change in pH.^32^ Moreover, precipitation began on the seventh day and gradually increased throughout the experiment, which also may explain the observed changes in pH. Considering all these data, the pH monitoring results confirmed the validity of the immersion tests, with questionable data for Al_2_O_3_^S^ only on the 28th day of the experiment. All data in detail is presented in Table S2 (see Supporting Information).

**Figure 5:**
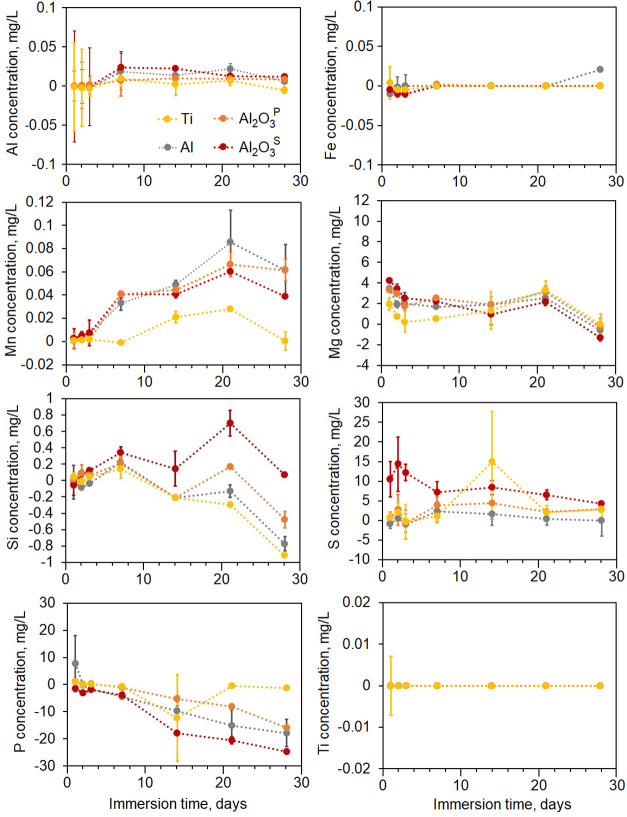
Total ion release of tested specimens after immersion in SBF for 1 to 28 days, determined by ICP-OES.

The pH curve was similar to previous studies that used an initial SBF solution with pH of 7.40 and hydroxyapatite/TiO_2_/Al_2_O_3_ nanocomposite coatings. The pH of the SBF solution increased to around 7.50 during the first days of immersion due to the accumulation of CO_3_^2–^, PO_4_^3–^ and OH^–^ ions.^49^ Researchers found that the accumulation of OH^–^ ions is essential for apatite nucleation. The deposition of Ca-P compounds, such as hydroxyapatite decreased the pH in some cases below the initial SBF values during longer immersion times.^50,51^ It is known that Al ions are toxic to the human body at high concentrations and may lead to local and systemic toxicity, inflammation, bone erosion, and implant rejection due to migration to surrounding tissues and organs.^52,53^ Therefore, the concentration in blood serum should not exceed >30 μg/L for Al alloy.^53^ The estimated daily intake of Al is much higher, i.e., 0.10–0.12 mg Al/kg/day for adults.^54^ Immersion tests showed no significant release of Al ions from tested alloys and coatings for up to 28 days in corrosive media due to surface passivation (Figure 5). The result is well correlated with sintered Al_2_O_3_ ceramics, which showed no release of Al ions after immersion in SBF for 14 days.^55^

Iron is one of the essential elements that regulates oxygen and electron transport, DNA synthesis, enzymatic reactions and other physiological processes. However, an excess of Fe ions is associated with lipid peroxidation and cell damage, which can lead to liver cancer, atherosclerosis, and hematological diseases.^56^ The normal concentration of Fe ions in blood serum ranges from 0.6 to 1.8 mg/L, while concentrations above 5 mg/L are considered toxic.^53^ There was no release of Fe ions into SBF from any of the tested specimens.

Another element of concern regarding the potential risk of neurotoxicity is Mn ions. They can accumulate in the brain and other tissues and organs following overexposure.^57^ The normal concentration of Mn in human tissues is approximately 1 mg/kg, while in blood serum, it ranges from 4 to 15 μg/L.^53^ The results indicated a gradual increase in Mn ions in SBF for both bioceramic coatings, particularly Al_2_O_3_^P^, due to its wider pores and higher surface porosity. However, the average experimental values did not exceed 0.1 mg/L. The estimated Mn values for Ti alloy were significantly lower compared to Al alloy and porous Al_2_O_3_. The maximum concentration of Mn ions were 0.061 and 0.067 mg/L for Al_2_O_3_^S^ and Al_2_O_3_^P^ coatings, respectively, and 0.086 mg/L for Al alloy after immersion in SBF for 28 days. These values are significantly lower compared to the glass-ceramic.^58^

The release of nontoxic Mg ions in the SBF solution was relatively consistent across all specimens, with the average concentration generally remaining below 4 mg/L.

Silicon (Si) is an essential trace element in the human body and plays an important role in the early stages of bone formation. Several studies demonstrated that Si promotes osteogenesis, cell adhesion, proliferation, and osteogenic differentiation, thereby improving osseointegration at the bone-to-implant interface.^59,60^ The release of Si tended to decrease for most tested specimens, particularly during longer immersion periods. The Al_2_O_3_^S^ samples exhibited a higher dissolution of Si ions; however, the maximum values did not exceed 1 mg/L. Nevertheless, the release of Si ions may be considered insignificant, as the desirable concentration for cellular response is expected to range from 30 to 52 mg/L.^61^

Phosphates play an important role in membrane integrity, skeletal biomineralization, homeostasis, and other vital functions of the body.^62^ Significant amounts of sulfates and phosphates are often incorporated into Al_2_O_3_ coatings during anodizing. In this study, the average concentrations of S and P in the coating were 3.90 and 1.00 atom %, respectively. The release of sulfates and phosphates from the pores of the anodic coating results in a higher total concentration of S and P ions at the beginning of immersion. The immersion tests demonstrated that the concentrations of S and P decrease with increasing immersion time on Al alloy and Al_2_O_3_ coatings. This decrease is due to precipitate formation resulting from the reaction of sulfates and phosphates with the Al alloy, which was visible in the SBF after 7 days. The reduction in P content is significantly greater during the 28 days, attributed to the formation of AlPO_4,_ which has much lower solubility in water than Al_2_(SO_4_)_3_ salt. On the other hand, the P content decrease is also attributed to the deposition of Ca–P compounds from the SBF solution onto Al_2_O_3_ coatings, as confirmed by previous studies.^50,63^ The release of sulfate anions was more pronounced in Al_2_O_3_^S^ samples during the initial immersion days, which is associated with an increase in body acidity.^64^

Ti is regarded as the most biocompatible metal due to its high corrosion resistance, ability to promote osseointegration, and mechanical strength. No release of Ti ions was observed. All data are summarized in Figure 5 and Table S3 (see Supporting Information).

### 3.3 Characterization of Bioceramic Coatings after SBF Immersion

**Figure 6:**
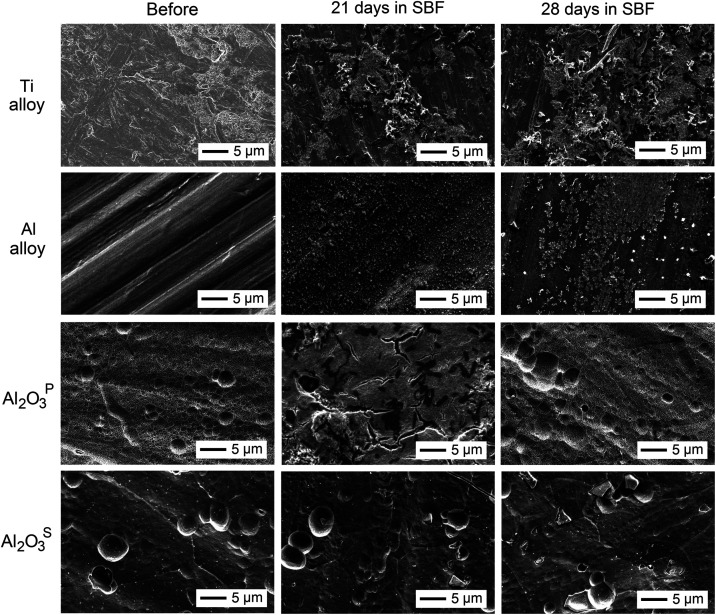
SEM topography of tested specimens.

Bone tissue is a complex composite material, primarily composed of organic collagen and inorganic calcium phosphate, such as hydroxyapatite (Ca_10_(PO_4_)_6_(OH)_2_), which naturally occurs in human bones and teeth. Hydroxyapatite obtained from SBF demonstrated high connectivity to bone tissues with a similar inorganic composition to natural bone.^65^ Surface topography plays an important part in mineral deposition. Rough surfaces with pores may enhance the adherence of deposited minerals and salts, thereby increasing osseointegration and the success of implantation.^66^ The surface topography of the specimens was evaluated after 21 and 28 days of immersion in SBF. Topographical studies revealed that the deposition of salts on the tested specimens varied in morphology and distribution after immersion in SBF for 21 and 28 days. Larger filaments formed on Ti alloys, while smaller pellets were observed on Al alloy with a tendency to coalesce after longer immersion durations (Figure 6). Large clusters with visible cracks and a size exceeding 50 μm were detected on Al_2_O_3_^P^ after 21 days of immersion. However, the adhesion of these clusters was poor, leading to their complete removal after 28 days. Surprisingly, no visible salts were detected on Al_2_O_3_^S^.

Surfaces with stoichiometric hydroxyapatite having a Ca/P ratio of 1.67, similar to that close of natural bone, are the most desirable.^66^ Hydroxyapatites formed from SBF typically exhibit a lower Ca/P ratio due to reduced crystallinity.^65^ EDS analysis was used to evaluate the content of deposited salts on the tested samples. According to EDS, the Ca/P ratio was highest for Ti alloy at 1.00 ± 0.12 and lowest for Al alloy at 0.33 ± 0.09. The bioceramic Al_2_O_3_ coatings demonstrated higher Ca/P ratios compared to Al alloy, specifically 0.39 ± 0.10 for Al_2_O_3_^P^ and 0.62 ± 0.06 for Al_2_O_3_^S^. Furthermore, the incorporation of negatively charged phosphates into the pores of the bioceramic coatings is much higher than that of Ca^2+^ cations suggesting that the specimens, suggesting that the specimens, such as Al_2_O_3_ coatings, may pass a positive charge on the surface and a negative charge at the bottom of the pores. This indicates that surface chemistry is an extremely important factor for bone mineralization around implants. Overall, apatites with distinct morphology were formed on all specimens, with relatively higher Ca/P ratios observed on Ti and bioceramic coatings compared to Al alloy. The alloys and coatings may require additional surface treatment to achieve bone-like hydroxyapatite with a higher Ca/P ratio.

### 3.4 Electrochemical Corrosion Studies of Al and Al_2_O_3_ Coatings

**Figure 7:**
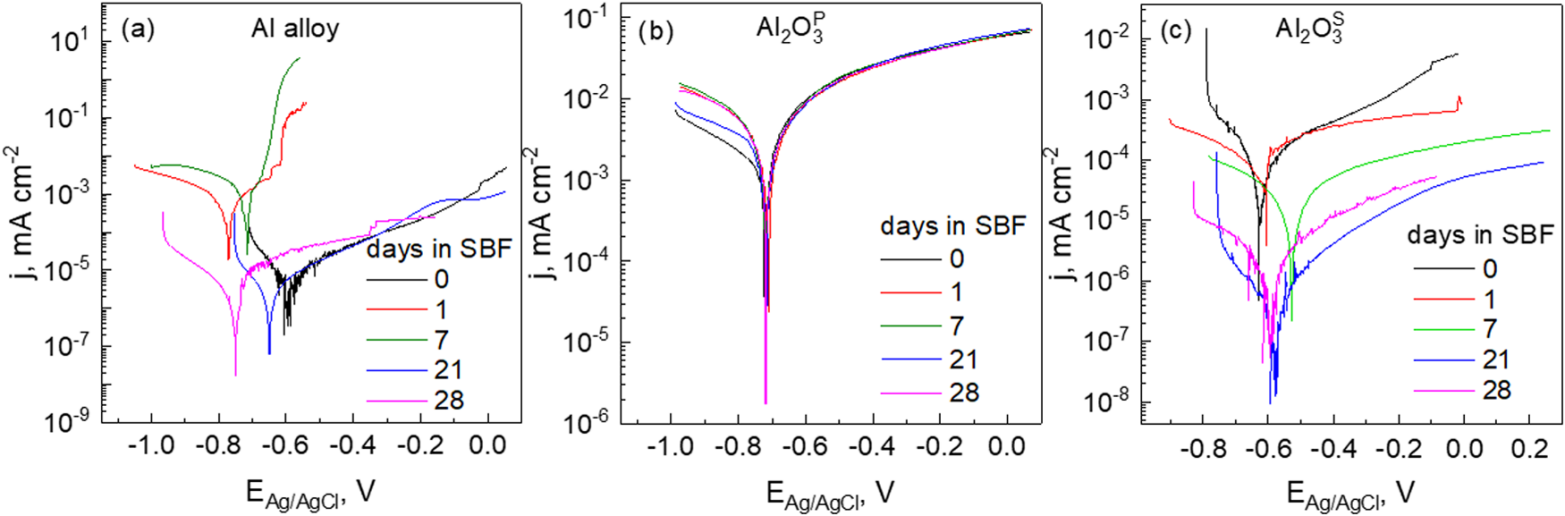
Tafel plots of (a) Al alloy, (b) Al_2_O_3_^P^ and (c) Al_2_O_3_^S^ before and after immersion in SBF for 1 to 28 days.

The Tafel plots for the unanodized Al alloy and the anodized Al_2_O_3_^P^ and Al_2_O_3_^S^ coatings in SBF over a 28-day immersion period are presented in Figure 7. Corrosion current densities (*j*_corr_) of the coatings were determined by extrapolation of the linear anodic and cathodic regions of the Tafel plot to the corrosion potential *E*_corr_ from the start of immersion to 28 days in SBF. It is known that higher positive *E*_corr_ and lower *j*_corr_ values indicate better corrosion resistance.^67^ The *R*_p_ value, defined as the polarization resistance, is determined by the slope of the voltammetric curve ±5 mV at the open circuit potential. This value is directly correlated with the corrosion rate; specifically, a higher *R*_p_ value indicates enhanced resistance of the coating to corrosion. The summarized electrochemical parameters are provided in Table 5.

**Table 5:** Electrochemical Corrosion Parameters of Al Alloy and Al_2_O_3_ Coatings

specimen	days in SBF	*E*_corr_, V	*j*_corr_, μA cm^–2^	*R*_p_, MΩ·cm^2^	corrosion rate, mm/year
Al alloy	0	–0.593	0.0027	10.50	14.6 × 10^–6^
	1	–0.769	0.2820	0.15	1.5 × 10^–3^
	7	–0.713	0.4260	0.05	2.3 × 10^–3^
	21	–0.648	0.0010	24.70	5.3 × 10^–6^
	28	–0.752	0.0012	17.30	6.8 × 10^–6^
Al_2_O_3_^P^	0	–0.704	1.2200	0.05	6.7 × 10^–3^
	1	–0.711	2.2500	0.04	24.6 × 10^–3^
	7	–0.719	2.1700	0.04	11.8 × 10^–3^
	21	–0.718	1.3100	0.05	7.2 × 10^–3^
	28	–0.719	1.4000	0.04	7.6 × 10^–3^
	0	–0.620	0.056	1.44	290 × 10^–6^
Al_2_O_3_^S^	1	–0.605	0.0570	1.40	285 × 10^–6^
	7	–0.528	0.0010	6.80	76.5 × 10^–6^
	21	–0.577	0.0003	177.00	1.4 × 10^–6^
	28	–0.594	0.0009	55.50	5.4 × 10^–6^

At the beginning of the experiment (day 0), the Al alloy exhibited an initial *E*_corr_ of −0.593 V, which was more positive than that of the anodized Al_2_O_3_^P^ (−0.704 V) and Al_2_O_3_^S^ (−0.620 V) coatings. The higher *E*_corr_ value of the Al alloy indicated the formation of a compact native oxide layer that initially provided corrosion protection. Furthermore, the relatively low *j*_corr_ of 0.0027 μA cm^–2^ indicated moderate initial corrosion resistance. However, with increasing immersion time (1–7 days), the *E*_corr_ shifted to a more negative value, reaching −0.752 V after 28 days. This shift was accompanied by a significant increase in *j*_corr_, which peaked at 0.426 μA cm^–2^ on day 7, while *R*_p_ decreased to 10.5 MΩ cm^2^. The observed decrease in E_cor_ and the increase in *j*_corr_ indicate accelerated corrosion, likely due to the presence of chloride (Cl^–^), phosphate (PO_4_^3–^) and sulfate (SO_4_^2–^) anions in the SBF solution, which affect the dissolution of the aluminum substrate. The repassivation effect was observed after 21 days, which was characterized by a decrease in *j*_corr_ to 0.001 μA cm^–2^ and an increase in *R*_p_ to 24.7 MΩ·cm^2^, suggesting the formation of a secondary protective layer, possibly composed of phosphate-based compounds from the SBF.

The corrosion behavior of the Al_2_O_3_^P^ coating was more stable throughout the immersion period. Initially, it exhibited the most negative *E*_corr_ of −0.704 V and the highest *j*_cor_ of 1.22 μA cm^–2^, indicating greater susceptibility to corrosion. However, its *E*_corr_ remained stable shifting slightly to −0.719 V, while *j*_cor_ minor exhibited minor fluctuations (1.4 μA cm^–2^ after 28 days) maintaining a steady state behavior. The enhanced stability of this coating was attributed to the dense oxide layer formed during the anodizing process. Furthermore, the interaction of Al_2_O_3_^P^ with SBF solution promoted the formation of hydroxyapatite-like layers, which may enhance its bioactivity.^67^

In contrast, the Al_2_O_3_^S^ coating exhibited superior corrosion resistance, as demonstrated by its Tafel plot. While, its initial *E*_corr_ of −0.620 V was more negative than that of Al alloy, it shifted progressively to more positive values, reaching – 0.594 V by day 28. This positive shift in *E*_corr_ was accompanied by a continuous decrease in *j*_cor_, from an initial 0.056 to 0.001 μA cm^–2^ by 7 day, while *R*_p_ increased from 6.80 to 177 MΩ·cm^2^ by day 21. The increase in *E*_corr_ and decrease in *j*_corr_ suggest the formation of a protective oxide layer, suppressing corrosion.

The initial corrosion rate of commercially pure titanium (CP-Ti) alloy was reported as 3.54 × 10^–2^ mm/year under similar testing conditions,^68^ which significantly exceeded that of the Al alloy (1.46 × 10^–5^ mm/year) and Al_2_O_3_^S^ (6.7 × 10^–3^ mm/year). While some studies have reported lower corrosion rates for etched CP-Ti (7 × 10^–4^ mm/year),^69^ the performance of Al_2_O_3_^S^ remained superior.

For comparison, Ahmadi and Afshar found that TiO_2_/Al_2_O_3_ reinforced hydroxyapatite sol–gel coatings were able to reduce corrosion current density from 0.42 to 0.091 μA/cm^2,^ depending on the amount of TiO_2_/Al_2_O_3_ composite.^50^ Improved corrosion resistance was related to the uniformity of sol–gel coatings without surface cracking. Another study showed that TiO_2_/hydroxyapatite nanocomposite with Ag and TiO_2_ nanoparticles applied on Ti6Al4V/TiO_2_ reduced corrosion current density to 0.22 μA/cm^2^ due to the lowest surface porosity and microcracking.^43^ The anticorrosion properties of previous findings were much better than porous Al_2_O_3_^P^ coatings, not only due to very low surface cracking and porosity, but also due to uniform Ca-P layer deposition from SBF. However, corrosion was still a few orders of magnitude lower than hard Al_2_O_3_^S^ coatings.

**Figure 8:**
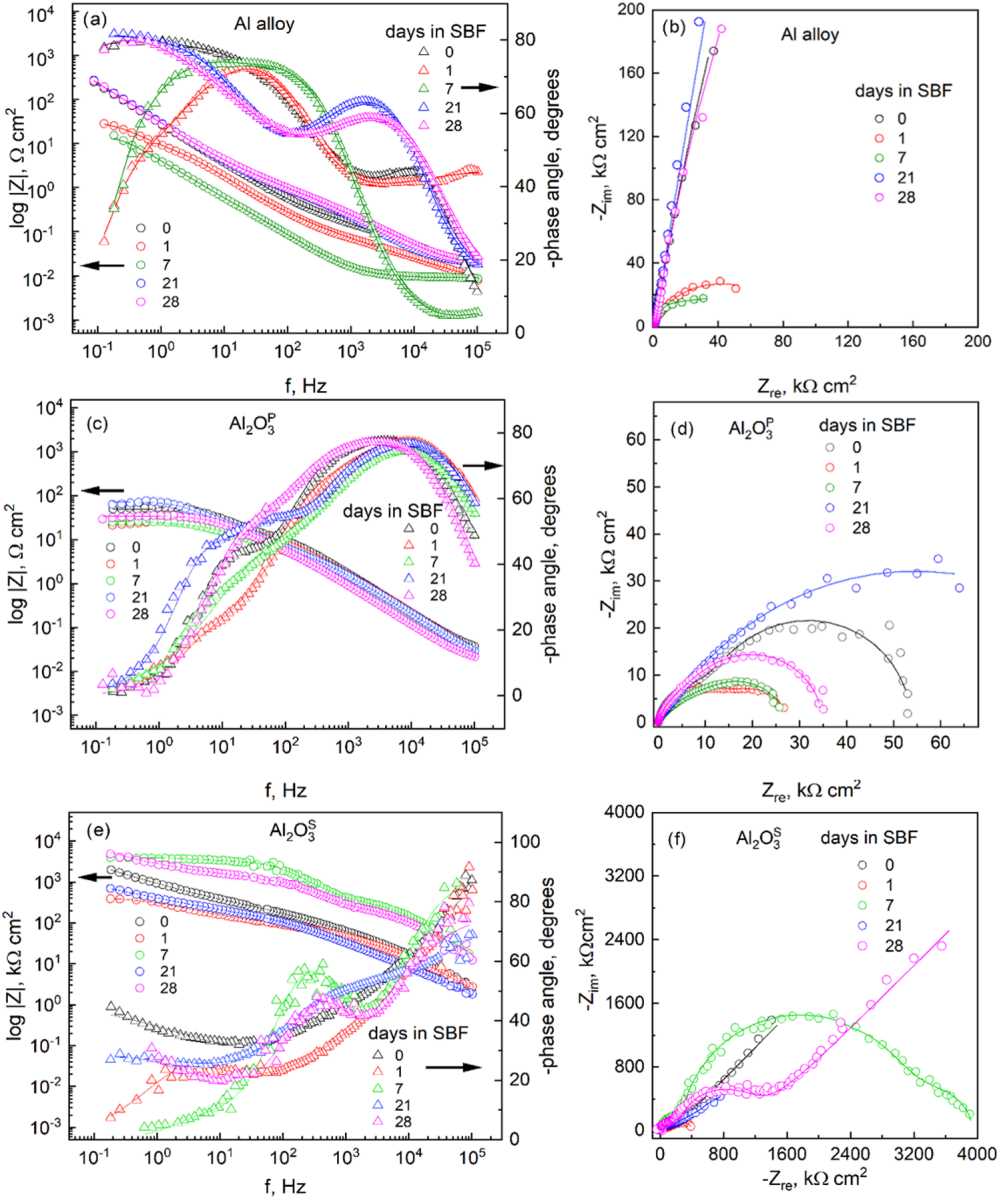
Bode (a, c, e) and Nyquist (b, d, f) plots of Al (a, b), Al_2_O_3_^P^ (c, d) and Al_2_O_3_^S^ (e, f) electrodes on exposure time in SBF solution.

The evaluation of the corrosion process parameters of untreated aluminum alloy and anodized aluminum in H_2_SO_4_ and H_3_PO_4_ was also performed by EIS analysis. The Bode and Nyquist plots of the untreated and anodized samples are shown in Figure 8. The analysis was performed over three frequency regions: high, mid and low, which were derived from the impedance spectra. The Bode phases of the studied electrodes are shown in Figure 8a,c,e. For the untreated Al alloy, the phase angle maximum initially reached ∼44° in the high frequency region and shifted to lower frequencies after 21 days, indicating the formation of passive oxide layers due to interaction with the SBF solution. In the midfrequency region, the second phase angle maximum increased to ∼70° after 7 days, indicating the formation of barrier oxide layers. These observations are closely related to the impedance modulus plot: the slope of the impedance dependence is −1, which is a characteristic response of a capacitive behavior of the surface film. For the Al_2_O_3_^P^ samples, the phase angle of ∼70° shifted to a higher frequency range during the first 7 days, indicating an increase in the capacitance of the primary formed anodic film. With longer immersion times, the phase angle shifted slightly to lower frequencies, indicating a repassivation film. In the case of the Al_2_O_3_^S^ samples, the initial phase angle of ∼80–90° measured in the high frequency range remains the same over the immersion time, indicating a highly passive electrode.

The impedance modulus Z provides a quantitative measure of the corrosion resistance of a metal/coating structure (Figure 8a,c,e). The anodized Al_2_O_3_^S^ exhibited the highest *Z* at low frequencies in comparison to the untreated Al alloy, indicating enhanced corrosion resistance and reduced alloy degradation. In the case of Al_2_O_3_^P^, the *Z* values remained similar to those of the untreated Al alloy during the initial immersion stages, but exhibited a slight increase over exposition time, suggesting a gradual improvement in corrosion resistance.

A comparison of the shape Nyquist plots (Figure 8b,d,f) reveals significant differences in the electrochemical behavior of untreated and anodized Al alloys at the metal/electrolyte interface. While the Nyquist plots of all electrodes are characterized by close semicircles in the high and the intermediate frequency range, and in low frequency ranges exhibit distinct characteristics of the real (*Z*_re_) and imaginary (*Z*_im_) parts, the Al_2_O_3_^S^ sample exhibited significantly higher values of the *Z*_re_ and *Z*_im_. Following a 28-day immersion period, the Nyquist plot revealed two overlapping semicircles at high and intermediate frequencies, indicating distinct electrochemical processes (Figure 8). As immersion continued, the differences in the shapes of the Nyquist plots became increasingly pronounced. The observed upward trend of *Z*_im_ across all samples, particularly in the mid- and low frequency ranges, indicates diffusion-controlled processes, likely due to ion transport through the porous (and corrosion product) layer. To quantitatively analyze the EIS data, an equivalent electrical circuit (EEC) was employed.

**Figure 9:**
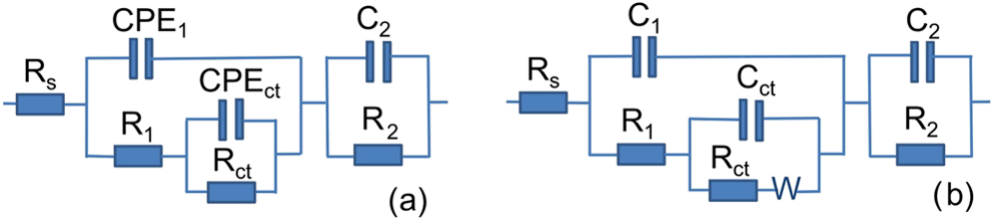
Equivalent electrical circuits used for fitting of EIS spectra of Al alloy and Al_2_O_3_^P^ (a), and Al_2_O_3_^S^ (b).

The evaluation of the impedance spectra using a Voigt circuit measurement model confirmed the presence of three time constants in the EIS spectra.^70^ The observed discrepancies in the impedance spectra of the Al alloy and anodized samples necessitated the implementation of two different types of EECs with an equivalent number of time constants to accurately fit the experimental data (Figure 9 (a) for Al alloy and Al_2_O_3_^P^ and (b) for Al_2_O_3_^S^). The proposed EEC is comprised of (RC) subcircuits arranged in series with *R*_s_, which represents the solution resistance between the working and reference electrodes. The high frequency time constant *R*_1_*C*_1_ is associated with the resistance and capacitance of the outer oxide layer in contact with the electrolyte. The second parallel time constant, observed at intermediate frequencies and represented by *R*_ct_*C*_dl,_ (the charge transfer resistance −Al corrosion reaction, and the double layer capacitance), is related to the more compact anodic layer located at the base of the pores. At low frequencies, the third time constant, *R*_2_*C*_2_, is attributed to a mass transport process, likely ion diffusion, within the porous/barrier layer on the metal substrate.

The Warburg (W) element was included in the EEC for the Al_2_O_3_^S^ alloy (Figure 9b) due to the presence of diffusion-controlled behavior at low frequencies. This suggests that ion diffusion through the passive layer or along the interface plays a key role in the overall electrochemical process. This behavior is consistent with the formation of a blocking phase - a thick, compact, and diffusion-limiting oxide film, which is a characteristic feature of the oxide growth mechanism.

In EEC (Figure 9a), the CPE_1_ parameter was used instead of pure *C*_1_ to account for the nonideal capacitive behavior caused by surface roughness and electrochemical inhomogeneity of the electrode.^71^ The fitted parameters are summarized in Tables 6, 7 and 8. Both EECs were characterized by the low values of the chi-squared parameter (χ^2^) and the weighted mean error values (*Z*_err_), indicating the quality of the fitting.

**Table 6:** Equivalent Electrical Circuit Fitting Data for Al Alloy Over Time in SBF Solution

days in SBF	*R*_s_ (Ω cm^2^)	CPE_1_ (Ss^n^ cm^–2^) (n)	*R*_1_ (Ω cm^2^)	CPE_ct_ (Ss^n^ cm^–2^) (n)	*R*_ct_ (Ω cm^2^)	*C*_2_ (F cm^–2^)	*R*_2_ (Ω cm^2^)	χ^2^ (*Z*_err,_ %)
0	19.6	7.43 × 10^–7^ (0.66)	122.8	2.27 × 10^–5^ (0.72)	1.39 × 10^5^	8.96 × 10^–6^	2.6 × 10^6^	4.48 × 10^–4^ (<2.1)
1	9.84	6.78 × 10^–6^ (0.89)	99.99	1.11 × 10^–5^ (0.95)	3.79 × 10^4^	3.50 × 10^–5^ (0.57)	172.2	7.63 × 10^–5^ (<0.83)
7	14.49	8.69 × 10^–5^ (0.59)	5.31	1.50 × 10^–5^ (0.91)	7.73 × 10^4^	3.23 × 10^–5^ (1)	1.58 × 10^4^	1.57 × 10^–4^ (<1.25)
21	17.86	2.81 × 10^–7^ (0.89)	951.3	4.55 × 10^–5^ (0.65)	1.18 × 10^5^	7.16 × 10^–6^	3.34 × 10^6^	3.05 × 10^–4^ (<1.75)
28	17.06	4.00 × 10^–6^ (0.89)	722.4	1.36 × 10^–5^ (0.54)	1.00 × 10^5^	7.36 × 10^–6^	1.27 × 10^6^	1.04 × 10^–4^ (<1.02)

**Table 7:** Equivalent Electrical Circuit Fitting Data for Al_2_O_3_^P^ Alloy Over Time in SBF Solution

days in SBF	*R*_s_ (Ω cm^2^)	CPE_1_ (Ss^n^ cm^–2^) (n)	*R*_1_ (Ω cm^–2^)	CPE_ct_, (Ss^n^ cm^–2^) (n)	*R*_ct_ (Ω cm^2^)	*C*_2_ (F cm^–2^)	*R*_2_ (Ω cm^2^)	χ^2^ (Z_err,_ %)
0	12.69	7.26 × 10^–8^ (0.89)	431.80	4.83 × 10^–7^ (0.72)	1.27 × 10^4^	6.68 × 10^–7^	4.06 × 10^4^	7.06 × 10^–4^ < 2.6
1	21.6	8.69 × 10^–7^ (0.67)	10.91	5.07 × 10^–8^ (0.99)	1.93 × 10^4^	5.19 × 10^–6^	8390	5.4 × 10^–4^ < 2.3
7	12.69	2.37 × 10^–6^ (0.63)	3869	2.55 × 10^–6^ (0.67)	1.21 × 10^4^	3.66 × 10^–6^	1.11 × 10^4^	1.0 × 10^–5^ < 1.0
21	12.3	9.68 × 10^–6^ (0.63)	3781	2.10 × 10^–6^ (0.99)	1.21 × 10^5^	3.36 × 10^–7^	1126	4.1 × 10^–4^ < 2.0
28	18.15	1.69 × 10^–7^ (0.99)	669	1.59 × 10^–7^ (0.98)	1.12 × 10^4^	5.73 × 10^–7^	2.39 × 10^4^	2.1 × 10^–4^ < 1.5

**Table 8:** Equivalent Electrical Circuit Fitting Data for f Al_2_O_3_^S^ Alloy Over Time in SBF Solution

days in SBF	*R*_s_ (Ω cm^2^)	*C*_1_ (F cm^–2^)	*R*_1_ (Ω cm^2^)	*C*_ct_ (F cm^–2^)	*R*_ct_ (Ω cm^2^)	*W* (Ss^–0.5^ cm^–2^)	*C*_2_ (F cm^–2^)	*R*_2_ (Ω cm^2^)	χ^2^ (Z_err,_ %)
0	49.45	4.47 × 10^–10^	4.56 × 10^5^	2.14 × 10^–8^	4.64 × 10^5^	1.36 × 10^–7^	1.67 × 10^–7^	1.3 × 10^6^	3.6 × 10^–3^ (<5.0)
1	55.21	4.89 × 10^–10^	1.63 × 10^5^	2.73 × 10^–8^	2.8 × 10^5^	1.60 × 10^–7^	5.42 × 10^–7^	1.98 × 10^5^	1.7 × 10^–3^ (<3.8)
7	73.28	1.36 × 10^–10^	1.59 × 10^6^	9.82 × 10^–10^	2.47 × 10^5^	1.58 × 10^–8^	8.55 × 10^–8^	3.52 × 10^5^	2.3 × 10^–4^ (<1.5)
21	43.50	7.26 × 10^–10^	7.81 × 10^5^	4.02 × 10^–7^	5.92 × 10^5^	3.59 × 10^–7^	4.22 × 10^–8^	1.60 × 10^4^	1.1 × 10^–3^ (<3.0)
28	51.34	2.34 × 10^–11^	2.81 × 10^6^	5.05 × 10^–10^	1.28 × 10^6^	1.29 × 10^–8^	2.62 × 10^–8^	4.16 × 10^6^	3.4 × 10^–3^ (<4.9)

The temporal evolution of the resistive (*R*) and capacitive (*C*) parameters provides detailed information on the electrochemical stability of the studied alloys. For the Al alloy, *R*_1_ increased from 122.8 to 951.3 Ω cm^2^ by day 21, followed by a decrease to 722.4 Ω cm^2^ by day 28. This indicates initial oxide growth followed by destabilization. Outer layer capacitance CPE_1_ increased significantly from 7.43 × 10^–7^ (day 0) to 8.69 × 10^–5^ S·s^n^·cm^–2^ (day 7), indicating increased surface activity or porosity, before sharply decreasing to 2.81 × 10^–7^ S·s^n^·cm^–2^ on day 21 with a partial recovery to 4.00 × 10^–5^ S·sn·cm^–2^ on day 28. The charge transfer resistance *R*_ct_ decreased sharply from 1.39 × 10^5^ to 3.79 × 10^4^ Ω cm^2^ on day 1 and then gradually increased to 1.00 × 10^5^ Ω cm^2^ on day 28, indicating decreased electrochemical stability. A gradual decrease in *R*_2_ (from 2.60 × 10^6^ to 1.27 × 10^6^ Ω cm^2^) and the associated C_2_ further support structural rearrangement within the inner oxide layer. These results indicate simultaneous processes of passivation, reorganization, and partial degradation of the passive film.

In the case of the Al_2_O_3_^P^ alloy, *R*_1_ was the highest on day 7 (3869 Ω cm^2^), but decreased to 669 Ω cm^2^ on day 28. A similar trend was observed for *R*_2_, which declined from 4.06 × 10^4^ to 2.39 × 10^4^ Ω cm^2^. While *R*_ct_ was 1.21 × 10^7^ Ω cm^2^ on day 7, it dropped to 1.12 × 10^6^ Ω cm^2^ on day 28, indicating reduced electrochemical stability. The capacitive elements, particularly CPE_1_, increased significantly from 7.26 × 10^–8^ to 1.69 × 10^–7^ S·s^n^·cm^–2^, suggesting an increase in surface porosity or inhomogeneity. These observations indicate unstable passive film behavior and a decline in corrosion resistance over time.

The Al_2_O_3_^S^ alloy showed the most stable electrochemical behavior. Both R_1_ and R_ct_ increased significantly, reaching 2.81 × 10^8^ and 5.05 × 10^7^ Ω cm^2^, respectively, on day 28. The capacitance components *C*_1_, *C*_dl_ and *C*_2_ consistently decreased, indicating increase in film compactness and a decrease in interfacial activity. The presence of a Warburg element throughout the period confirms the contribution of diffusion-controlled charge transport, which is characteristic of dense barrier-type oxides. These trends indicate that Al_2_O_3_^S^ exhibits a stable, capacitive, and diffusion-limited passive film, resulting in improved corrosion resistance.

The fitted parameters also allow for the evaluation of the polarization resistance *R*_p_, which is inversely proportional to *j*_cor_. The *R*_p_ value is calculated as the sum of *R*_1_, *R*_ct_ and *R*_2_. The Al_2_O_3_^S^ samples exhibited the highest *R*_p_ values or the lowest *j*_cor_ values during all immersion times, thereby demonstrating their superior corrosion resistance.

### 3.5 Biocompatibility Test

**Figure 10:**
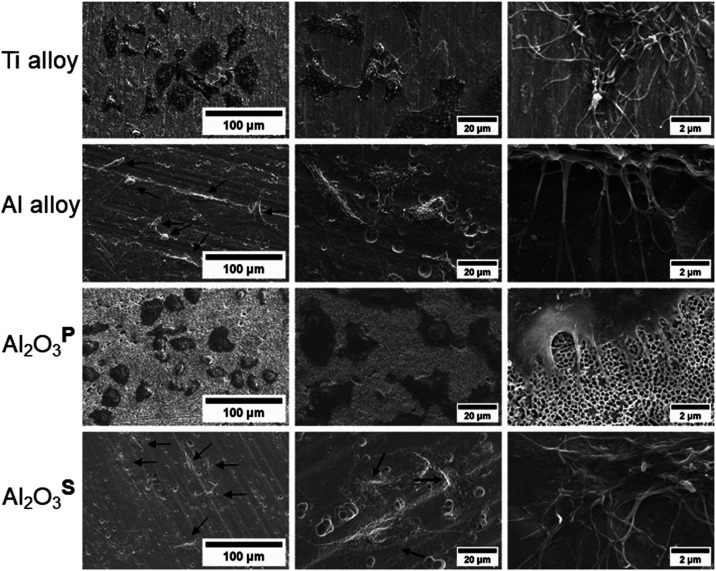
SEM images of L929 cells cultured on Ti alloy, Al alloy, Al_2_O_3_^P^, and Al_2_O_3_^S^ samples at various magnifications. Arrows indicate cells with low contrast.

The growth of L929 cells on the surfaces of the specimens was visualized using SEM (Figure 10). A small number of cells were selected to highlight their direct contact with the surfaces. The Ti and Al_2_O_3_^P^ samples demonstrated a high abundance of cells. In contrast, the Al alloy and Al_2_O_3_^S^ samples exhibited minimal contrast in the SEM images. This made cell quantification less discernible, although not necessarily low. The cells on the Ti alloy, Al alloy and Al_2_O_3_^S^ specimens appeared uniform in nature. The morphology and filopodia of the cells on these specimens were characterized by two types: elongated and flattened, both of which are indicative of healthy cells. Furthermore, both filopodia and extracellular matrix (ECM) expression were clearly observed.

In contrast, the cells on Al_2_O_3_^P^ displayed only a flattened morphology. This flattening is attributed to the topography and pore size of the surface.^18,72^ Additionally, the filopodia on Al_2_O_3_^P^ were challenging to observe. Although visible at the base of the cells, the protruding parts were obscured between or within the pores. This remains an assumption as visualizing the interior of the pores is difficult, likely due to complex contrast. Despite these features, the L929 cells were visibly attached and appeared to have spread across the surface.

From an adhesion perspective, cells exhibiting extended and flattened morphologies suggest that the surface facilitates strong adhesion and promotes active cellular processes, such as spreading and migration. This was observed with the Al_2_O_3_^S^ coating. In contrast, the Al_2_O_3_^P^ coating demonstrated a flattened morphology of L929 cells. While this indicates that the surfaces support cell adhesion, it may not effectively promote processes such as spreading and migration, and could potentially interfere with cell viability.

The cytotoxicity of the alloys was assessed by evaluating the viability of L929 fibroblasts exposed to alloy extracts prepared using a culture medium. The analysis focused on two key aspects: (a) the overall effect on cell growth, including adhesion and subsequent proliferation, and (b) the effect on proliferation alone.

**Figure 11:**
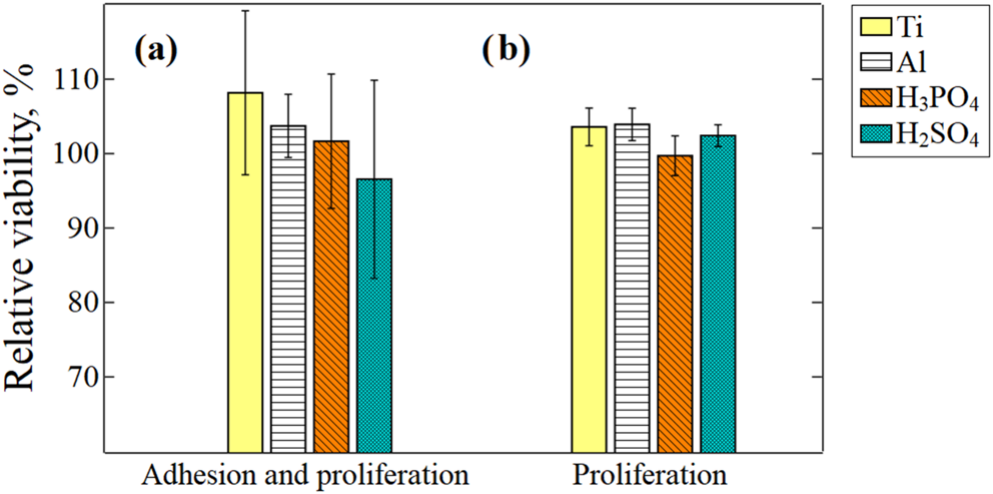
Impact of alloy extracts on the relative viability of L929 cells: (a) overall growth (including adhesion and proliferation) and (b) proliferation alone (excluding adhesion) The mean relative viability (%) was calculated based on the untreated control group, with error bars indicating the standard deviation. No significant differences were observed between groups in both (a, b) (one-way ANOVA, *p* = 0.61 and *p* = 0.66, respectively).

The initial stages of interaction between cells and biomaterials include attachment, adhesion, and proliferation. The effectiveness of these early phases significantly affects overall growth. To examine the effects on both adhesion and subsequent proliferation, L929 cells were detached, suspended in the extracts of the specimens, and seeded onto a tissue-treated plate for a 24 h incubation period. Cell viability was assessed by measuring mitochondrial activity using the resazurin assay. The relative viability (%) of cells treated with different extracts was as follows: 108.2 ± 11.0% (Ti), 103.7±4.3% (Al alloy), 101.7 ± 9.0% (Al_2_O_3_^P^), and 96.6 ± 13.3% (Al_2_O_3_^S^) (Figure 11a).

To evaluate the influence of the specimen on (b) cell proliferation, excluding adhesion, L929 cells were initially allowed to adhere in a monolayer before the growth media was substituted with the extracts. After 24 h of culturing with the extracts, cell viability was assessed by measuring mitochondrial activity using the XTT assay (Figure 11b). The relative viability (%) of cells treated with different extracts was as follows: 105.5 ± 2.5% (Ti), 101.4 ± 2.2% (Al alloy), 99.4 ± 2.7% (Al_2_O_3_^P^), and 101.5 ± 1.5% (Al_2_O_3_^S^).

In both experiments, one-way ANOVA analysis revealed no statistically significant differences between the specimens in either analysis: (a) overall growth and (b) proliferation only (*p* = 0.61 and *p* = 0.66, respectively). The greater scatter of the error bars in (a) can be attributed to experimental conditions and the instruments used. Thus, no substantial disparities were observed among the tested alloy types regarding their impact on cell viability.

In summary, the quantitative viability assay indicated a noncytotoxic nature for all specimens, with relative cell viability exceeding 95% after incubation with extracts. The cell adhesion demonstrated effective attachment to the surface of the specimens, with filopodia and ECM expressed. The micropores and higher porosity on the Al_2_O_3_^P^ surface resulted in a flattened cell morphology, which may indicate interference with cellular processes. In contrast, the nanopores and lower porosity of Al_2_O_3_^S^ confirmed the suitability of its surface topographical features for successful cell culturing.

### 3.6 Summary

Biocompatibility is closely associated with the surface structure of biomaterials and the release of metal ions, both of which can significantly impact cell adhesion and viability. For instance, TiO_2_/Al_2_O_3_ reinforced hydroxyapatite coatings deposited on stainless steel 316L increased cell viability to 99.5%. In comparison, hydroxyapatite coatings on stainless steel and bare stainless steel exhibited reduced viability rates of 75 and 51%, respectively, after 72 h of seeding.^50^ This reduction is attributed to the high surface cracking and porosity of hydroxyapatite coatings, which expose the stainless steel to SBF, resulting in corrosion and potentially releasing toxic Fe ions. In another study involving TiO_2_/hydroxyapatite nanocomposite with the presence of TiO_2_ and Ag nanoparticles applied on Ti6Al4V/TiO_2_ reported an acceptable cell viability of 98.8%. This improvement is due to enhanced coating uniformity with a crack-free structure and suppression, which mitigates migration of toxic Al and V ions.^43^ Additionally, research indicated that a Ti6Al4V alloy combined with a 20% ZrO_2_ ceramic composite positively influenced biocompatibility, resulting in increased cell viability by up to 98.25%. In comparison, a hybrid composite of ZrO_2_ and hydroxyapatite demonstrated the highest cell viability, recorded at 107.53%.^73^ Detailed analyses suggest that bioceramic Al_2_O_3_ coatings produced through a one-step Al anodizing process offer superior cell viability and biocompatibility compared to composite ceramics.

This study investigated the biocompatibility of Al alloy 6082 and its porous bioceramic Al_2_O_3_ coatings, obtained through anodization of Al alloy 6082 using two different electrolytes: phosphoric acid and sulfuric acid. The evaluation focused on corrosion resistance, ion leakage from the alloys, cell growth and cytotoxicity, with results experimentally compared to a medical-grade Ti alloy.

The Al_2_O_3_^S^ bioceramic coating, developed through anodization with sulfuric acid, demonstrated several significant attributes related to its biocompatibility. The Al_2_O_3_^S^ coating effectively suppressed the release of Fe and toxic Al ions. This inhibition is critical as the elevated concentration of Al ions would lead to toxicity, inflammation, or implant rejection. Additionally, Mn ion release from Al_2_O_3_^S^ was measured at approximately 0.04 mg/L, which remains within safe limits (below the 0.1 mg/L threshold established in other studies). Furthermore, there was a notable increase in the release of sulfate ions, which may contribute to increased acidity in the body. Mg and Si ions, essential for various physiological functions, were released in nontoxic quantities, supporting the viability of surrounding cells. Moreover, a higher dissolution of Si promotes osteogenesis, cell adhesion and proliferation, thereby enhancing osseointegration.

The corrosion resistance following a 28-day immersion of the specimen in SBF demonstrated that Al_2_O_3_^S^ exhibited superior corrosion resistance, compared to the original Al alloy and Al_2_O_3_^P^. The corrosion rate of Al_2_O_3_^S^ was over 100 times lower than that of compared to CP-Ti.^68^ The high corrosion resistance of Al_2_O_3_^S^ is advantageous, as it ensures the stability and longevity of biomaterials, thereby supporting the viability of cells in contact with it. The reduced corrosion rates diminish the likelihood of harmful ion release. Prolonged exposure to SBF further enhanced corrosion resistance, contributing to increased stability.

Al_2_O_3_^S^ maintains the lightweight characteristics of the original Al alloy, while its hardness is comparable to that of Ti alloy, making it highly desirable for implant applications. The bioceramic coating of Al_2_O_3_^S^ features nanopores that facilitate a supportive environment for cellular activities. Fibroblast cells cultured on Al_2_O_3_^S^ displayed both extended and flattened morphologies, with viability experiments confirming these findings.

The biocompatibility of Al_2_O_3_^S^ is underscored by its enhanced stability, hardness, and supportive cellular environment, rendering it highly suitable for implantation. However, the elevated migration of sulfates necessitates careful monitoring to ensure it does not negatively impact the local tissue environment.

Another bioceramic coating, Al_2_O_3_^P^, produced by anodizing Al alloy with phosphoric acid, displayed distinct characteristics. Similar to Al_2_O_3_^S^, the Al_2_O_3_^P^ coating also effectively suppressed the release of Fe and Al ions. However, the release of Mn ions from Al_2_O_3_^P^ was measured at approximately 0.06 mg/L, which is still within the safe limits but higher than the release from Al_2_O_3_^S^. In terms of corrosion, Al_2_O_3_^P^ bioceramic coatings demonstrated the highest corrosion rate and reaction with SBF, which is not a desirable characteristic for biomaterials. However, its property is beneficial for the formation of a hydroxyapatite layer, which is advantageous for bone bonding. Similar to Al_2_O_3_^S^, longer exposure to SBF resulted in increased corrosion resistance, contributing to enhanced stability.

In contrast to Al_2_O_3_^S^, the hardness of the lightweight Al_2_O_3_^P^ was reduced, attributed to micropores and increased surface porosity. Cells on Al_2_O_3_^P^ exhibited only the flattened morphology, potentially due to the larger pores in the coating. This may interfere with cellular processes such as spreading and migration, impacting viability. However, viability experiments did not reveal any negative effect in the short term.

The uncoated Al alloy 6028 also demonstrated good ion release properties. Similar to the bioceramic coatings, the Al alloy effectively inhibited the release of toxic Al and Fe ions. However, the release of Mn ions was the highest among all specimens. In terms of corrosion resistance, the Al alloy demonstrated adequate performance, although it did not match the stability and durability of the anodized coatings. Cell viability tests on fibroblast cells revealed no significant differences in cell viability or cytotoxicity compared to anodized specimens or Ti alloy. The adhesion pattern was similar to that of Ti alloy and Al_2_O_3_^S^.

In conclusion, the data indicate that among the tested specimens, the Al_2_O_3_^S^ bioceramic coating emerges as the most promising material for biomedical applications. It is important to note that these experimental results are based on short-term and static conditions. In clinical applications, biomaterials are subjected to various physiological forces, including mechanical stress, fluid flow, and dynamic interactions with surrounding tissues. These stresses can lead to mechanical damage or surface scratches, potentially compromising the integrity of the anodized layer and exposing the underlying Al alloy. To better reflect clinical scenarios, future studies should focus on longer durations of experiments and mechanical testing to evaluate potential damage and enhance the corrosion resistance of biomaterials.

## 4 Conclusions

We investigated the chemical stability and biocompatibility of Al alloy 6082 and two types of porous bioceramic Al_2_O_3_ coatings, produced by anodizing the Al alloy 6082 with phosphoric or sulfuric acid electrolytes. Both treated and untreated specimens effectively inhibited the release of Fe and toxic Al ions. However, the untreated Al alloy exhibited a higher release of Mn ions compared to the coated specimens. Furthermore, both the Al alloys and bioceramic coatings exhibited excellent viability with the fibroblast cell line. The fibroblast cells adhered to the Al alloy and Al_2_O_3_^S^ with the extended and flattened morphology, while cells on Al_2_O_3_^P^ displayed only flattened morphology. The Al_2_O_3_^S^ bioceramic coating exhibited hardness comparable to Ti alloy and demonstrated remarkable biocompatibility along with the highest corrosion resistance among all specimens. Therefore, we suggest Al_2_O_3_^S^ for biomedical applications, although further biomedical research is necessary.

## Supporting Information Available

Characteristics of surface pores of anodic coatings (Table S1); influence of immersion time on pH of SBF and tested specimens (Table S2); total ion release of tested specimens after immersion in SBF for 1 to 28 days, determined by ICP-OES (Table S3) (PDF)
