# Supplementary material for: Corrosion and Biocompatibility Studies of Bioceramic Alumina Coatings on Aluminum Alloy 6082
Source: ACS Appl Mater Interfaces. 2025 Apr 18;17(17):24901–17. doi: 10.1021/acsami.5c00532 (PMC12051176; doi:10.1021/acsami.5c00532)
Supplement: Supplementary file 1 — am5c00532_si_001.pdf [file am5c00532_si_001.pdf]

Supporting Information

Corrosion and biocompatibility studies of bioceramic alumina coatings on aluminum alloy 6082

Tadas Matijosius[*$1,2], *Neringa Bakute*[*$3], *Juozas Padgurskas*[1], *Ausra Selskiene*[4], *Aleksej Zarkov*[6], *Asta Griguceviciene*[5], *Justina Kavaliauskaite*[3], *Arunas Stirke*[3] *and Svajus Joseph Asadauskas*[2,†]

[1]*Faculty of Engineering, Vytautas Magnus University (VMU), Studentu 15, LT 53362 Akademija, Kaunas district, Lithuania*

[2]*Department of Chemical Engineering and Technology, State Research Institute Center for Physical Sciences and Technology, Sauletekio 3, LT 10257 Vilnius, Lithuania*

[3]*Department of Functional Materials and Electronics, State Research Institute Center for Physical Sciences and Technology, Sauletekio 3, LT 10257 Vilnius, Lithuania*

[4]*Department of Characterisation of Materials Structure, State Research Institute Center for Physical Sciences and Technology, Sauletekio 3, LT 10257 Vilnius, Lithuania*

[5]*Department of Electrochemical Material Science, State Research Institute Center for Physical Sciences and Technology, Sauletekio 3, LT 10257 Vilnius, Lithuania*

[6]*Institute of Chemistry, Vilnius University, Naugarduko 24, LT-03225 Vilnius, Lithuania*

* Tadas Matijosius, e-mail: tadas.matijosius@vdu.lt, tadas.matijosius@ftmc.lt

* Neringa Bakute, e-mail: neringa.bakute@ftmc.lt

[$] T.M. and N.B. contributed equally to this paper.

[†] In memoriam. We are deeply saddened by his passing, and his memory will continue to inspire us.

Table S1. Characteristics of surface pores of anodic coatings.

Specimen	Electrolyte	Total pore area, μm^2	Total surface area, μm^2	Pore number	Pore diameter, nm	Pore density, pores/μm^2	Porosity, %
$Al_2O_3^S$	H_2SO_4	0.033	0.25	223	13.8	892.0	13.4
		0.031	0.25	210	13.6	840.0	12.2
		0.037	0.25	251	13.8	1004.0	14.9
$Al_2O_3^P$	H_3PO_4	0.498	1.00	19	182.7	19.0	49.8
		0.450	1.00	16	189.2	16.0	45.0
		0.481	1.00	24	159.7	24.0	48.1

Table S2. Influence of immersion time on pH of SBF and tested specimens.

Immersion time, days	SBF	Al	$Al_2O_3^S$	$Al_2O_3^P$	Ti
1	7.43±0.04	7.42±0.02	7.40±0.03	7.40±0.02	7.41±0.02
2	7.41±0.01	7.45±0.05	7.41±0.01	7.39±0.00	7.42±0.00
3	7.48±0.01	7.44±0.06	7.48±0.01	7.48±0.01	7.48±0.02
7	7.49±0.05	7.50±0.08	7.49±0.10	7.48±0.10	7.43±0.07
14	7.47±0.04	7.49±0.03	7.49±0.06	7.45±0.01	7.37±0.05
21	7.47±0.00	7.46±0.02	7.44±0.00	7.43±0.02	7.46±0.00
28	7.29±0.16	7.48±0.02	7.29±0.09	7.43±0.01	7.34±0.03

Table S3. Total ion release of tested specimens after immersion in SBF for 1 to 28 days, determined by ICP-OES.

Immersion time, days	Al concentration, mg/L			
	Al	$Al_2O_3^S$	$Al_2O_3^P$	Ti
1	-0.001±0.004	0.000±0.071	0.000±0.019	-0.001±0.057
2	-0.001±0.001	0.000±0.021	0.001±0.029	-0.002±0.049
3	-0.002±0.001	-0.001±0.049	0.001±0.011	-0.003±0.009
7	0.019±0.023	0.024±0.020	0.007±0.020	0.010±0.010
14	0.014±0.001	0.023±0.002	0.010±0.006	0.002±0.014
21	0.022±0.006	0.013±0.006	0.009±0.005	0.008±0.007
28	0.006±0.000	0.012±0.000	0.008±0.005	-0.005±0.004
Immersion time, days	Fe concentration, mg/L			
	Al	$Al_2O_3^S$	$Al_2O_3^P$	Ti
1	-0.010±0.000	-0.005±0.007	-0.005±0.007	0.004±0.020
2	-0.001±0.013	-0.010±0.000	-0.010±0.000	-0.005±0.007
3	0.000±0.014	-0.010±0.000	-0.010±0.000	-0.005±0.007
7	0.000±0.000	0.000±0.000	0.002±0.003	0.000±0.000
14	0.000±0.000	0.000±0.000	0.000±0.000	0.000±0.000
21	0.000±0.000	0.000±0.000	0.000±0.000	0.000±0.000
28	0.021±0.000	0.000±0.000	0.000±0.000	0.000±0.000
Immersion time, days	Mg concentration, mg/L			
	Al	$Al_2O_3^S$	$Al_2O_3^P$	Ti
1	3.420±0.332	4.210±0.163	3.335±0.212	1.940±0.615
2	1.920±0.339	3.415±0.488	2.945±0.106	0.740±0.071
3	2.005±0.700	2.515±0.559	1.735±0.233	0.230±0.990
7	1.670±0.106	2.205±0.057	2.525±0.255	0.515±0.014
14	1.910±0.742	0.920±0.997	1.895±0.042	1.310±1.831
21	2.540±0.417	2.155±0.354	3.110±0.629	3.315±0.863
28	-0.580±1.103	-1.340±0.046	-0.270±0.806	0.000±0.976
Immersion time, days	Mn concentration, mg/L			
	Al	$Al_2O_3^S$	$Al_2O_3^P$	Ti
1	0.002±0.000	0.003±0.008	0.001±0.007	0.001±0.000
2	0.005±0.004	0.005±0.002	0.001±0.000	0.001±0.000
3	0.007±0.000	0.008±0.011	0.002±0.000	0.002±0.000

7	0.033±0.006	0.041±0.003	0.041±0.001	-0.001±0.001
14	0.049±0.004	0.041±0.003	0.045±0.006	0.021±0.005
21	0.086±0.028	0.061±0.001	0.067±0.011	0.028±0.000
28	0.062±0.022	0.039±0.000	0.062±0.009	0.001±0.008

Immersion time, days	Si concentration, mg/L			
	Al	$Al_2O_3^S$	$Al_2O_3^P$	Ti
1	-0.020±0.205	-0.060±0.120	0.015±0.057	0.040±0.049
2	-0.085±0.021	0.080±0.042	0.090±0.099	-0.020±0.099
3	-0.035±0.014	0.125±0.000	0.065±0.057	0.045±0.071
7	0.225±0.007	0.340±0.071	0.210±0.085	0.145±0.120
14	-0.211±0.000	0.143±0.212	-0.211±0.000	-0.211±0.000
21	-0.131±0.078	0.701±0.153	0.172±0.010	-0.297±0.012
28	-0.774±0.088	0.069±0.000	-0.479±0.098	-0.916±0.013

Immersion time, days	P concentration, mg/L			
	Al	$Al_2O_3^S$	$Al_2O_3^P$	Ti
1	7.775±10.324	-1.415±0.693	1.100±0.361	0.895±0.042
2	-0.250±0.028	-3.055±0.177	0.070±0.255	-0.195±0.106
3	-1.635±0.665	-1.895±0.042	0.375±0.467	0.115±0.156
7	-4.460±0.028	-3.975±1.068	-1.210±0.311	-0.690±0.396
14	-9.760±1.959	-17.865±0.212	-5.225±1.259	-12.385±15.952
21	-15.115±5.869	-20.555±1.329	-8.195±0.750	-0.450±0.516
28	-17.885±4.999	-24.790±0.431	-15.930±0.028	-1.355±0.728

Immersion time, days	S concentration, mg/L			
	Al	$Al_2O_3^S$	$Al_2O_3^P$	Ti
1	-0.905±1.082	10.535±4.504	0.620±1.457	0.710±1.117
2	0.555±0.806	14.330±6.979	2.725±3.875	2.025±2.333
3	-0.980±3.656	12.230±2.058	-0.630±2.369	-0.350±4.434
7	2.285±2.454	7.230±2.687	3.790±2.206	1.095±1.803
14	1.640±2.906	8.365±1.853	4.475±4.016	14.995±12.813
21	0.335±1.534	6.540±1.259	2.175±1.648	2.020±1.796
28	-0.090±3.847	4.220±0.000	2.820±0.523	2.685±2.242

Immersion time, days	Ti concentration, mg/L			
	Al	$Al_2O_3^S$	$Al_2O_3^P$	Ti
1	0.000±0.000	0.000±0.000	0.000±0.000	0.000±0.007

2	0.000±0.000	0.000±0.000	0.000±0.000	0.000±0.000
3	0.000±0.000	0.000±0.000	0.000±0.000	0.000±0.000
7	0.000±0.000	0.000±0.000	0.000±0.000	0.000±0.000
14	0.000±0.000	0.000±0.000	0.000±0.000	0.000±0.000
21	0.000±0.000	0.000±0.000	0.000±0.000	0.000±0.000
28	0.000±0.000	0.000±0.000	0.000±0.000	0.000±0.000
